# Deep Clonal Profiling of Formalin Fixed Paraffin Embedded Clinical Samples

**DOI:** 10.1371/journal.pone.0050586

**Published:** 2012-11-30

**Authors:** Tara Holley, Elizabeth Lenkiewicz, Lisa Evers, Waibhav Tembe, Christian Ruiz, Joel R. Gsponer, Cyrill A. Rentsch, Lukas Bubendorf, Mark Stapleton, Doug Amorese, Christophe Legendre, Heather E. Cunliffe, Ann E. McCullough, Barbara Pockaj, David Craig, John Carpten, Daniel Von Hoff, Christine Iacobuzio-Donahue, Michael T. Barrett

**Affiliations:** 1 Clinical Translational Research Division, Translational Genomics Research Institute, Scottsdale, Arizona, United States of America; 2 Collaborative Bioinformatics Center, Translational Genomics Research Institute, Phoenix, Arizona, United States of America; 3 Institute for Pathology, University Hospital Basel, University of Basel, Basel, Switzerland; 4 Department of Urology, University Hospital Basel, University of Basel, Basel, Switzerland; 5 NuGEN, San Carlos, California, United States of America; 6 Computational Biology Division, Translational Genomics Research Institute, Phoenix, Arizona, United States of America; 7 Department of Laboratory Medicine, Mayo Clinic, Scottsdale, Arizona, United States of America; 8 Department of Surgery, Mayo Clinic, Scottsdale, Arizona, United States of America; 9 Neurogenomics Division, Translational Genomics Research Institute, Phoenix, Arizona, United States of America; 10 Integrated Cancer Genomics Division, Translational Genomics Research Institute, Phoenix, Arizona, United States of America; 11 Virginia G. Piper Cancer Center, Scottsdale Healthcare, Scottsdale, Arizona, United States of America; 12 Johns Hopkins University, Baltimore, Maryland, United States of America; National Cancer Institute, National Institutes of Health, United States of America

## Abstract

Formalin fixed paraffin embedded (FFPE) tissues are a vast resource of annotated clinical samples. As such, they represent highly desirable and informative materials for the application of high definition genomics for improved patient management and to advance the development of personalized therapeutics. However, a limitation of FFPE tissues is the variable quality of DNA extracted for analyses. Furthermore, admixtures of non-tumor and polyclonal neoplastic cell populations limit the number of biopsies that can be studied and make it difficult to define cancer genomes in patient samples. To exploit these valuable tissues we applied flow cytometry-based methods to isolate pure populations of tumor cell nuclei from FFPE tissues and developed a methodology compatible with oligonucleotide array CGH and whole exome sequencing analyses. These were used to profile a variety of tumors (breast, brain, bladder, ovarian and pancreas) including the genomes and exomes of matching fresh frozen and FFPE pancreatic adenocarcinoma samples.

## Introduction

Formalin fixed paraffin embedded (FFPE) tissues are a vast resource of clinically annotated samples with patient follow-up data. As such, these samples represent highly desirable and informative materials for the application of high definition genomics that could improve patient management and provide a molecular basis for the selection of personalized therapeutics. The development of whole exome and whole genome technologies provides an unparalleled opportunity for advances in improved treatment and diagnosis for patients with cancer [1], [2]. One major limitation to the use of routinely prepared FFPE tissues is the highly variable and typically poor quality of the DNA extracted from samples of interest [3]–[6]. In addition high-resolution genomic analyses of biomaterials from human specimens are highly dependent on the cellular composition of the specimens [7], [8]. For example, a high degree of surrounding normal cells in a tumor biopsy can make it difficult to isolate a sufficient number of neoplastic cells for analysis of cancer genomes with a high degree of sensitivity [8]–[10]. Recent studies have described various methods to interrogate FFPE samples with array and sequencing technologies. These typically select samples exceeding a threshold for tumor cell content based on histological methods such as evaluation of H&E stained slides and macrodissection prior to analysis [11]. Once selected samples are processed in bulk using various protocols consisting of dewaxing, removal of protein crosslinks, followed by DNA extraction and purification [12], [13]. However, many samples, notably tumors arising in solid tissues exhibit high degrees of tissue heterogeneity, with varied admixtures of reactive stroma, inflammatory cells and necrosis in immediate contact with tumor cells. Thus, histology-based processes including laser capture microdissection (LCM) can be time consuming and labor intensive when purifying tumor cells from non-tumor cells in complex biopsies. Consequently, current approaches for the analyses of cancer genomes using FFPE samples are limited in their ability to advance translational genomics for improving patient management and clinical outcomes.

In order to optimize high definition genomic analysis of FFPE samples we used DNA content based assays to identify and sort nuclei of diploid and aneuploid populations from a variety of archived tissues. We optimized DNA extraction and amplification protocols to provide templates suitable for aCGH and whole exome mutational analysis by next generation sequencing (NGS) of flow sorted FFPE tumor populations. This included matching fresh frozen (FF) and FFPE pancreatic ductal adenocarcinoma (PDA) samples that were used to assess our ability to profile the genomes of this highly lethal cancer using archived samples. We subsequently interrogated FFPE samples from a variety of solid tumor tissues, including triple negative breast carcinomas (TNBCs), glioblastomas, bladder carcinoma, and small cell carcinoma of the ovary, to validate our methods. Finally we used matching FF and FFPE samples from a rapid autopsy PDA sample, and a matching primary cell line with a previously published exome sequence, to validate the use of sorted FFPE samples for NGS analysis [10]. The high definition genomic profiling of objectively defined highly purified populations of tumor cells from FFPE samples has broad application for cancer research and for advancing more personalized therapies for cancer patients.

**Figure 1:**
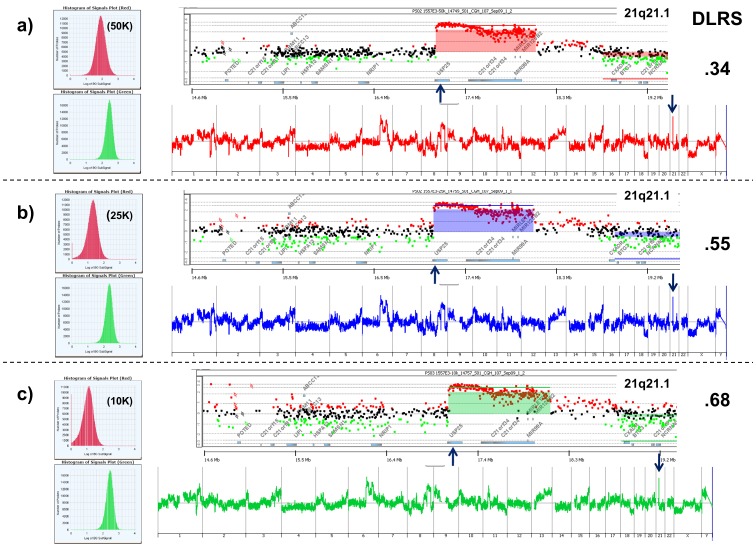
Sorted nuclei from FFPE samples required for aCGH. Signal intensity histograms (left), gene and whole genome aCGH plots (middle), and the derivative log ratio spreads (DLRS) (right) for hybridizations done with varying inputs from a sorted FFPE triple negative breast cancer sample PS02 1557 E3. A) 50,000 sorted nuclei input for DNA extraction and Cy-5 labeling (red channel in histogram). B) 25,000 sorted nuclei input for DNA extraction and Cy-5 labeling (red channel in histogram). C) 10,000 sorted nuclei input for DNA extraction and Cy-5 labeling (red channel in histogram). Shaded areas in aCGH plots denote ADM2-defined copy number aberrant regions. The gene view shows a focal amplicon that disrupts the *USP25* locus. A pooled 46,XX sample was used as a Cy-3 labeled (green channel in histogram) reference for each hybridization.

## Methods

### Clinical Samples

PDA samples were obtained under a WIRB protocol (20040832) for an NIH funded biospecimen repository (NCI P01 Grant CA109552) and with approved consent of the Ethics Committee of Basel (252/08, 302/09).The SCCO samples were collected under WIRB protocol 20101205. All fresh frozen samples were snap frozen in liquid nitrogen at the time of collection then stored at −80°C until processing for sorting according to our published protocols [14]. All tumor samples were histopathologically evaluated prior to analysis.

**Figure 2:**
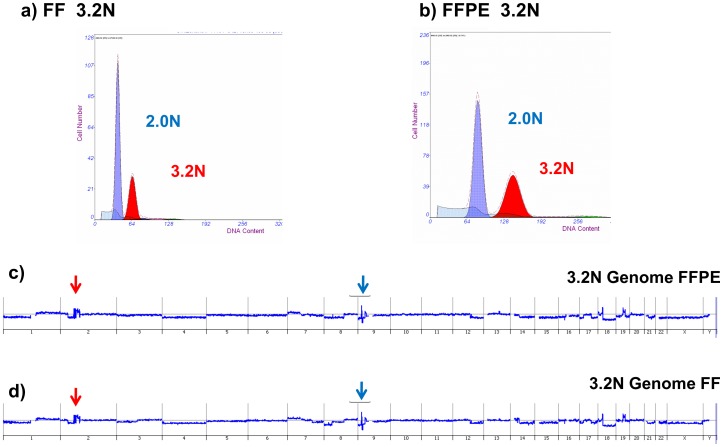
Whole genome comparison of aCGH results with matching sorted FFPE and FF samples. Flow sorting and aCGH analysis of matching fresh frozen (FF) and formalin fixed paraffin embedded (FFPE) samples from a pancreatic ductal adenocarcinoma. Flow sorting histogram and detection of 3.2N tumor population in A) FF tissue 11164 and B) FFPE tissue B3733. C–D) Whole genome aCGH plots of 3.2N population sorted from each tissue. Red arrow denotes highly aberrant region on chromosome 2. Black arrow denotes highly aberrant region on chromosome 9. Light blue shaded areas in lower left of A and B show sample and cell debris in flow data.

**Figure 3:**
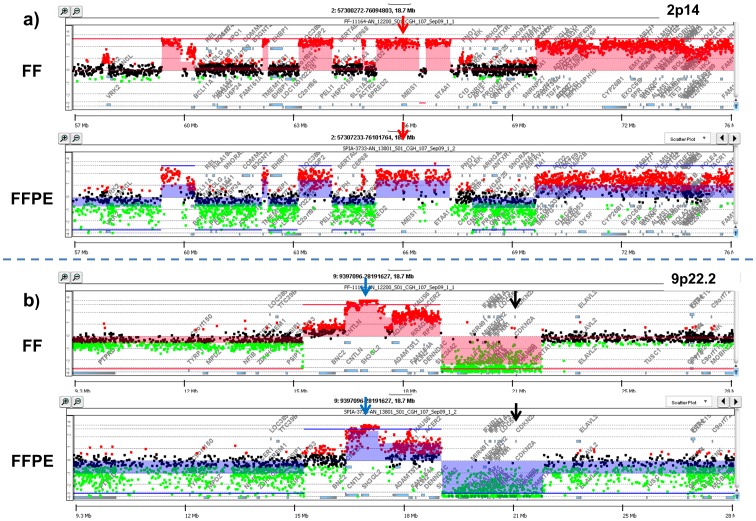
Gene-specific comparison of aCGH results with matching sorted FFPE and FF samples. Gene view comparison of ADM2 calls in matched fresh frozen (FF) and formalin fixed paraffin embedded (FFPE) pancreatic ductal adenocarcinoma sample. A) Chromosome 2p14 region includes a focal amplicon with the *MEIS1* gene (arrow). B) Chromosome 9p22.2 region includes a homozygous deletion of *CDKN2A* (black arrow) and a focal amplicon of *SH3GL2* (blue arrow). Shaded areas denote ADM2 defined copy number aberrant region.

### FFPE Sample Preparation and Flow Sorting

FFPE samples were fixed in formalin at the time of collection then stored according to routine pathology methods. Prior to sorting excess paraffin was removed with a scalpel from either side of 40–60 um scrolls to reduce accumulation of debris during the sorting process. Each scroll was collected into individual microcentrifuge tubes then washed three times with 1 ml Xylene for 5 minutes to remove remaining paraffin. Each sample was rehydrated in sequential ethanol washes (100% 5 minutes x2, then 95%, 70%, 50% and 30% ethanol) and washed 2 times in 1 ml 1 mM EDTA pH 8.0. A 1 ml aliquot of 1 mM EDTA pH 8.0 was added to the samples and incubated at 95°C for 80 minutes to facilitate the removal of protein cross-links present in FFPE tissue. Samples were then cooled to room temperature for >5 minutes, followed by addition of 300 µl PBS pH 7.4 and gentle centrifugation for 2 minutes at 3.6×g. The supernatant was carefully removed and the pellet washed three times with 1 ml PBS pH 7.4/0.5 mM CaCl_2_ to remove EDTA. Each sample was digested overnight (6–17 hours) in 1 ml of a freshly prepared enzymatic cocktail containing 50 units/ml of collagenase type 3, 80 units/ml of purified collagenase, and 100 units/ml of hyaluronidase in PBS pH 7.4/0.5 mM CaCl_2_ buffer. Each enzyme was rehydrated with PBS pH 7.4/0.5 mM CaCl_2_ buffer then stored at −20°C immediately prior to addition to the cocktail mixture. Following overnight digestion 500 µl NST was added to each sample to facilitate pelleting. Samples were centrifuged for 5 minutes at 3000×g, after which pellets were resuspended in 750 µl of NST/10% fetal bovine serum and then passed through a 25 G needle 10–20 times. The samples were filtered through a 35 um mesh and collected into a 5 ml Polypropylene round bottom tube. The mesh was rinsed with an additional 750 µl of NST/10% fetal bovine serum and placed on ice while processing remaining samples. The total volume in the tube for each sample was approximately 1.5 ml. An equal volume of 20 µg/ml DAPI was added to each tube to achieve a final concentration of 10 µg/ml DAPI prior to flow sorting with a BD Influx cytometer with ultraviolet excitation (Becton-Dickinson, San Jose, CA). The optimal settings for sorting FFPE samples with the Influx sorter were as follows: Drop formation was achieved with piezzo amplitude of 6–10 volts and a drop frequency of 30 khertz. The sort mode was set to purity yield with a drop delay of 31.5 32. Sheath fluid pressure was typically 17–18 psi with a 100 µm nozzle. For single parameter DNA content assays DAPI emission was collected at >450 nm. In each sorting experiment we used a single 50 µm FFPE scroll to obtain sufficient numbers of intact nuclei for subsequent molecular assays. DNA content and cell cycle were then analyzed using the software program MultiCycle (Phoenix Flow Systems, San Diego, CA).

**Figure 4:**
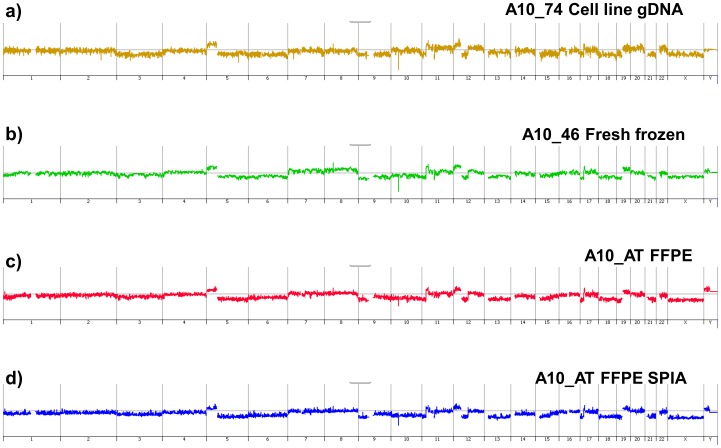
Whole genome aCGH plots of matching cell line and sorted aneuploid pancreatic ductal adenocarcinoma samples. A) Cell line A10_74 genomic DNA. B) Flow sorted 3.0N tumor population from fresh frozen tissue phi29 amplified DNA. C) Flow sorted 3.0N tumor population from formalin fixed paraffin embedded tissue (FFPE) and genomic DNA. D) Flow sorted 3.0N tumor population from FFPE tissue SPIA amplified DNA.

**Figure 5:**
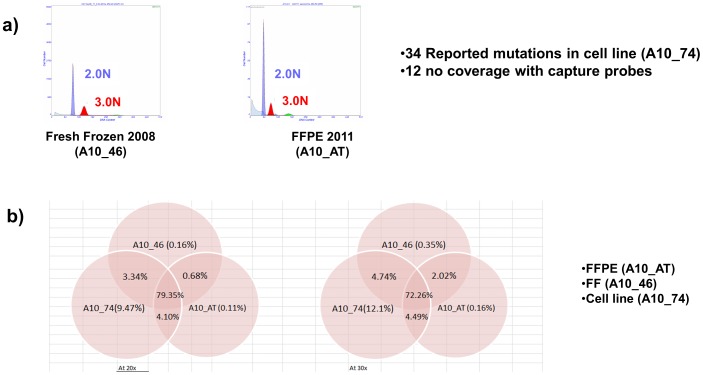
Whole exome sequencing of matching cell line and sorted FF and FFPE PDA tissues. A) Flow cytometry histograms and the detection of a 3.0N tumor population in FF (A10-46) and FFPE (A10_AT) tissues. B) Exomic sequencing coverage for 3.0N PDA genome from flow sorted FF (A10-46) and FFPE (A10_AT) tissues, and the matched tumor derived cell line A10_74. Left >20×coverage, right >30×coverage. The % coverage is based on Agilent SureSelect target regions.

**Figure 6:**
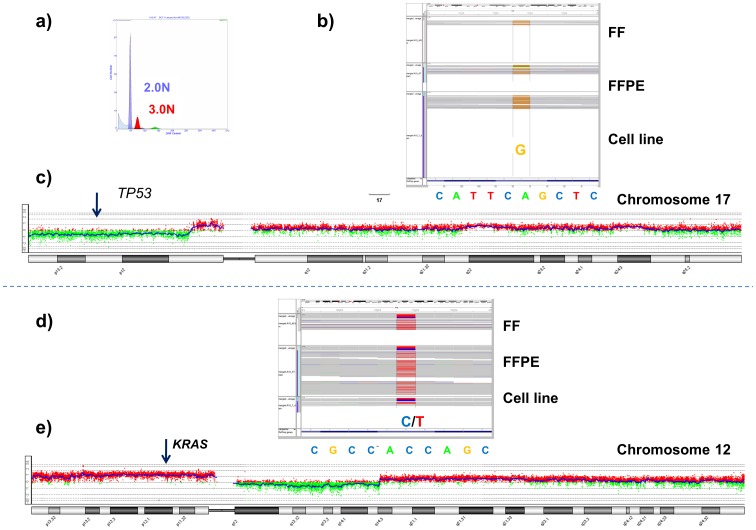
Combined aCGH and whole exome analysis. Whole exome sequencing of sorted fresh frozen (FF) and formalin fixed paraffin embedded (FFPE) pancreatic ductal adenocarcinoma (PDA) tissue. A) Flow sorted 3.0N tumor population from PDA tissue. B) Homozygous *TP53* mutation in sorted FF and FFPE tissues, and matching cell line. C) Chromosome 17 CGH plot of 3.0N population from sorted FF sample. D) Heterozygous *KRAS* mutation in sorted FF and FFPE tissues, and matching cell line. E) Chromosome 12 aCGH plot of 3.0N population from sorted FF sample.

### DNA Extraction

DNA from sorted nuclei was extracted using an amended protocol from QIAamp® DNA Micro Kit from Qiagen (Valencia, CA). Briefly each sorted sample was resuspended in 180 µl buffer ATL and 20 µl proteinase K then incubated for 3 hours at 56°C for complete lysis. Samples were bound and washed according to QIAamp® DNA Micro Kit instructions, eluted into 50 µl of H_2_0, then precipitated overnight with 5 µl 3 M sodium acetate and 180 µl 100% EtOH. Each sample was then centrifuged for 30 minutes at 20,000×g, washed in 1 ml of 70% EtOH for 30 minutes at 20,000×g. The samples were carefully decanted and the DNA pellet was dried by speed vacuum then resuspended in a small volume (e.g. 10–50 µl) of H_2_O for final concentrations suitable for accurate quantification.

### DNA Amplification

Genomic DNAs from sorted FFPE samples were amplified using Ovation® WGA FFPE System from NuGEN® Technologies (San Carlos, CA). DNA was processed in accordance with Ovation® WGA FFPE standard SPIA protocol with an alternate T7 endonuclease fragmentation step. Resulting amplified product was either used as template for aCGH analysis or processed with the Nugen Encore ds-DNA module according to the supplier’s instructions in order to generate double-stranded (ds) end repaired DNA as input for library suitable for next generation sequencing. Extracted fresh frozen sourced genomic DNA was amplified using the phi29 based Illustra GenomiPhi V2 Amplification kit from GE Healthcare Bio-sciences Corp (Piscataway,NJ) according to our published protocols [14]. A 100 ng aliquot of pooled 46, XX DNA (Promega, Madison, WI) was amplified with the matching amplification protocol to generate a suitable reference for each aCGH experiment using amplified DNA template. In all cases the quality of the amplification product was assessed by gel electrophoresis.

### aCGH Analysis

Fresh frozen phi29 amplified and FFPE non-amplified DNAs were treated with DNAse 1 prior to Klenow based labeling. High molecular weight phi29 templates were digested for 30 minutes while the smaller fragmented FFPE samples were digested for only 1 minute. In each case 1 µl of 10× DNase 1 reaction buffer and 2 µl of DNase 1 dilution buffer were added to 7 µl of DNA sample and incubated at room temperature then transferred to 70°C for 30 minutes to deactivate DNase 1. In contrast the amplified FFPE sourced DNAs do not require DNase 1 treatment prior to Klenow-based labeling. Sample and reference templates were then labeled with Cy-5 dUTP and Cy-3 dUTP respectively using a BioPrime labeling kit (Invitrogen, Carlsbad, CA) according to our published protocols [14]. All labeling reactions were assessed using a Nanodrop assay (Nanodrop, Wilmington, DE) prior to mixing and hybridization to 400 k CGH arrays (Agilent Technologies, Santa Clara, CA) for 40 hours in a rotating 65°C oven. All microarray slides were scanned using an Agilent 2565C DNA scanner and the images were analyzed with Agilent Feature Extraction version 10.7 using default settings. The aCGH data was assessed with a series of QC metrics then analyzed using an aberration detection algorithm (ADM2) [15]. The latter identifies all aberrant intervals in a given sample with consistently high or low log ratios based on the statistical score derived from the average normalized log ratios of all probes in the genomic interval multiplied by the square root of the number of these probes. This score represents the deviation of the average of the normalized log ratios from its expected value of zero and is proportional to the height h (absolute average log ratio) of the genomic interval, and to the square root of the number of probes in the interval. All aCGH data in this paper have been deposited at the National Center for Biotechnology Information Gene Expression Omnibus accession number GSE40299.

### Exome Library Preparation

3 µg of high quality genomic DNA with a 260/280 ratio between 1.8 and 2.1 was fragmented to a target size of 150 to 200 bp on the Covaris E210 system. Fragmentation was verified on a 2% TAE gel and fragmented samples were end-repaired using New England Biolab’s NEB Next kit (Ipswich, MA). Repaired samples were adenylated at the 3′ end using the NEBNext kit, and Illumina indexed adapters were next ligated onto A-tailed products. Samples were next PCR amplified using Herculase II polymerase and purified. Samples were then run on an Agilent Bioanalyzer (specify which chip) to verify amplification and to quantify samples. Samples were adjusted to 147 ng/µL for 24 hour hybridization to exonic RNA probes using Agilent’s SureSelect All Exon 50 Mb Plus kit, which contains 561,823 probes targeting 202,124 exons. Captured products were next selected for, purified, and PCR amplified. Final libraries were verified and quantified using an Agilent Bioanalyzer.

### Paired End Next Generation Sequencing

Libraries were denatured using 2N NaOH and diluted with HT2 buffer (Illumina). 1% of denatured and diluted phiX was spiked into each lane to allow for error rate reporting on the HiSeq. Cluster generation was performed using Illumina’s cBot and HiSeq Paired End Cluster Generation Kit. Flow cells were paired end sequenced on Illumina’s HiSeq 2000 using Illumina’s HiSeq Sequencing Kit. Raw sequencing data were converted to standard FASTQ format using CASAVA pipeline with in-house custom scripts [16], [17]. FASTQC program was used for quality control and all reads were trimmed to 90 high-quality base pairs. In order to generate at least 100 million pass filter reads for each exome library, 2 lanes of a HiSeq 2000 flowcell were sequenced for each of the FFPE and fresh frozen exomes, whereas only 1 lane was needed for the cell line exome. Overall, 130 million pass filter reads were generated for the fresh frozen sample, 190 million pass filter reads for the FFPE sample, and 192 million pass filter reads for the cell line sample. Data was aligned to hg18 assembly of human genome using BWA sequence alignment software (version 0.5.9) and raw alignment BAM files were further processed for quality recalibration, duplicate removal and local realignment using a custom in-house pipeline based on Picard and GATK tools [18]–[21]. The alignment statistics are summarized in Tables S1 and S2. For each sample, variants were called from BAM files using samtools and varscan using a minimum coverage cut-off of 10, and only those variants that were called by both algorithms were retained [22], [23].

### Fluorescence in-situ Hybridization

Fluorescence in-situ hybridizations (FISH) were performed as previously described [14]. Hybridization and post-hybridization washes were done according to the ‘LSI procedure’ (Vysis). Hybridizations with the 9p21 (ZytoLight SPEC p16/CEN9 Dual probe, Zytovision) and the Cyclin D1 (ZytoLight SPEC CCND1/CEN11 Dual probe, Zytovision) FISH probes were performed overnight in a humidified chamber at 37°C. All FISH analyses were independently evaluated by two people. Images were obtained by use of an Axioskop 40 fluorescence microscope (Zeiss) equipped with a 63× objective and an Axiocam MRm camera (Zeiss).

## Results

### Flow Sorting of Tumor Populations from Archived FFPE Samples

DNA content based flow assays can discriminate cell/nuclei populations based on ploidy including diploid, aneuploid, and elevated 4N(G_2_/M tetraploid) fractions from fresh frozen biopsies of interest [24]. These assays can be combined with tissue and tumor specific markers to sort subpopulations of diploid and aneuploid populations from routinely collected samples [25]–[27]. Our previous studies have shown that sorted populations provide optimal templates for high resolution detection of somatic aberrations in each cancer genome [14]. For example homozygous deletions can be detected in aCGH experiments using rigorous objective thresholds (log_2_ratios <−3.0) even in samples with high admixtures (>90%) of non-tumor cells. To apply these methods for FFPE samples, thick sections (40–60 µm) were initially de waxed, rehydrated in sequential ethanol washes, treated with EDTA then processed with a cocktail of collagenases and hyaluronidase to obtain single nuclei suspensions suitable for flow sorting. For each sample the nuclei were stained with 4,6′-diamidino-2-phenylindole, dihydrochloride (DAPI), disaggregated, and then filtered immediately before analyses on an Influx cytometer (Becton-Dickinson, San Jose CA), with ultraviolet excitation and DAPI emission collected at >450 nm. The flow rates were typically less than 1000 events/second and were adjusted accordingly for each sample based on sorting efficiency, the size and width of each peak of interest, and the presence of variable amounts of debris. DNA content and cell cycle fractions for each sorted population were analyzed, as previously described [14].

### Sorted FFPE Input for aCGH

To determine the number of sorted FFPE nuclei needed for robust aCGH results we sorted duplicate aliquots of 10,000, 25,000, and 50,000 diploid and aneuploid (3.2N) nuclei from a TNBC sample and processed the DNAs for hybridization to 60 mer oligonucleotide CGH arrays (Figure S1A–B). All hybridizations were done with a pooled commercial 46, XX reference. To assess the utility of sorted FFPE samples for aCGH analysis we compared a series of metrics including background subtracted dye normalized signal intensities, the standard deviation of the log ratio differences between consecutive probes across all chromosomes (dLRsd) for each experiment, and the ability to map aberrant intervals in each genome (Figure 1). The signal intensities of the sorted TNBC samples increased in a linear manner with increasing number of nuclei. We obtained robust signals relative to the reference channel using 50,000 sorted nuclei from the FFPE specimen. This increased signal resulted in a corresponding decrease in the dLRsd and improved resolution for aberration detection using a step gram algorithm ADM2 [15]. For example although high level (log_2_ ratio >1) amplicons such as one targeting the ubiquitin specific peptidase 25 (*USP25)* locus on 21q21.1 were detected and mapped in each hybridization, the weaker signals and broader distribution of ratios resulted in the progressive loss of detection of lower level amplicons, deletions, and the mapping of break points (Figure S1C–D). Significantly a homozygous deletion in tumor necrosis factor alpha-induced protein 8 (*TNFAIP8*), a negative mediator of apoptosis, was only detected in the 50,000 nuclei sample.

To further evaluate the use of sorted solid tissue FFPE samples we selected PDA samples with matching FF material. We sorted a minimum of 50,000 aneuploid and diploid nuclei from the FFPE samples and a minimum of 10,000 nuclei from each population in the matching FF samples (Figure 2). The width of the histograms for the diploid and aneuploid (3.2N) peaks was greater for the FFPE sample likely reflecting the lower quality of the sample relative to the FF sample. DNA from the sorted FF sample was amplified using our established phi29 methods [14]. A 1 ug aliquot of the amplification reaction was used for DNAse 1 digestion, labeling, and hybridization. In contrast unamplified low molecular weight DNAs extracted from the FFPE nuclei were used to prepare labeled templates. After hybridization and feature extraction we used the ADM2 intervals to measure the reproducibility of aCGH data in the matching FFPE and FF samples. Two intervals were called similar if their genomic regions overlapped by more than 0.5. The overlap of two intervals is defined as the genomic length of their intersection divided by the genomic length of their union. We selected the top 20 ranked amplicons in the FFPE sample for this analysis. In 19 of these 20 amplicons the overlap was >0.9 with the same ADM2-defined interval in the sorted fresh frozen sample. These intervals included a series of focal amplicons on chromosomes 2 and 9 that highlighted known and putative oncogenes (Figure 3). One striking example was a highly focal amplicon that targeted a single gene, *BCL11A*, and was detected in both matching samples.

We then assessed the global utility of our FFPE assays with different tissues including TNBCs, bladder carcinoma, glioblastoma, and small cell carcinoma of the ovary (SCCO) (Figures S2, S3, S4, S5, S6) and verified selected aberrations by FISH (Figure S7). These samples were obtained from multiple tumor banks and contained variable amounts of debris and non-tumor cells. We used single parameter DNA content assays to detect and sort the diploid, aneuploid, and 4N cell populations present in each sample. In each case we were able to discriminate homozygous and partial deletions, and map breakpoints and amplicon boundaries to the single gene level in the sorted samples regardless of tumor cell content. These include potentially clinically relevant aberrations such as focal amplicons of *EGFR*, *USP25*, and *CCND1*, and homozygous deletions in *PARD3*, *CDKN2A*, and *PTEN*. These latter aberrations included single exon deletions. One striking exception was SCCO a rare tumor that presents in very young women and girls [28]. The SCCO genomes did not contain any focal amplicons or homozygous deletions. However the resolution of our assays with FFPE samples allowed us to map a 1p36.22 breakpoint created by a single copy loss to the *CASZ1* locus, a zinc finger gene implicated in neuroblastoma [29] (Figure S6).

### Sorted FFPE Input for NGS

Current NGS protocols typically require larger amounts of genomic DNA template as input. Furthermore widely used methods preselect samples with high (e.g. >70%) tumor content and are dependent on genomic DNA templates of highly uniform quality as inputs for library construction [8], [30]. The small fragment sizes of DNAs typically isolated from routine FFPE samples are not suited for linear amplification with highly processive enzymes such as phi29. Therefore we investigated the use of single primer isothermal amplification (SPIA) (NuGEN Ovation) to generate templates from sorted FFPE samples that are suitable for aCGH and NGS. To rigorously test this method we compared aCGH data from matching FF, non-amplified FFPE, and SPIA FFPE samples. We collected aliquots of 10,000, 25,000 and 50,000 nuclei during sorts of individual pancreas FFPE samples. Each sorted aliquot was extracted, amplified, labeled, and then hybridized to 400 k CGH arrays. In each case the amplified product labeled to high specific activity. The amplified DNA from 50,000 nuclei samples gave robust signals on the array as measured by the histogram of the dye normalized background subtracted signals in the sample (Cy-5) channel (Figure S8). In contrast there was a second non-specific peak in the aCGH data obtained with the lower input samples. This suggests that non-specific products were generated in the amplification reaction that although they labeled efficiently did not hybridize to the unique human sequences of the CGH probes. These also correlated with the broadening of the distribution of the log_2_ ratios and the decreasing resolution in the detection of the aberrant genomic intervals in each genome. In contrast the ADM2-defined CGH intervals from the amplified 50,000 nuclei template matched those from the unamplified template as well as the FF sample (Figures S9, S10).

To assess SPIA-amplified sorted FFPE samples for NGS we resorted 50,000 nuclei from a FFPE PDA sample for which we also had matching FF sorted sample, and a PDA cell line (A10-74) whose exome has been previously reported [10]. We repeated the SPIA amplification with 50,000 FFPE nuclei input. Amplified products were then processed with the NuGen Encore ds-DNA module to generate double-stranded end repaired DNA as input for libraries suitable for NGS. This process typically required 1 to 2 weeks from accessing the FFPE sample to generating the final dsDNA input for NGS. We also prepared template for sequencing by amplifying 100 ng of genomic DNA from the sorted FF sample with our phi29 protocol, and from 3 µg of unamplified genomic DNA extracted from the cell line. In contrast to FFPE tissue samples these typically required half the time for preparing dsDNA templates for NGS. The genome profiles of the 3 samples, including the amplified FFPE derived DNA before and after the ds-DNA module, were identical as assessed by ADM2 intervals and the ploidy of the tumor cells (Figure 4).Separate 3 µg aliquots of SPIA-amplified dsDNA FFPE, phi29 amplified FF, and cell line genomic DNA were then used as inputs for exome sampling and NGS library preparations.

A comparison of the paired end reads alignments against the reference genome in each of the 3 samples showed that almost 80% of the target areas had at least 20× coverage in all three samples (Figure 5). The 34 known non-synonymous mutations were compared across the 3 samples. In twelve cases the regions of interest were not targeted by the capture oligonucleotides. For the remaining 22 mutations, a total of 62 variants were observed across the 3 samples. The 4 absent variants mapped to 2 loci that were not called in both the sorted FF and FFPE samples (Table S1). In one case (chromosome 19) the coverage in the sorted samples was very low (<10) compared to the cell line. Although the coverage for the second loci (chromosome 6) was also lower in these samples only the reference allele was called in 15 (FFPE) and 19 (FF) reads for a variant detected in 42% of the reads in the cell line. This discrepancy in variant calling as well as the low levels (24–35%) of reference alleles on chromosome 3 in the sorted samples could also be affected by allelic variation (“drift”) in the cell line or the presence of non- tumor cells in the sorted samples. However given the sort profiles of the 3.0N population in the FF and FFPE samples, the concordance of the majority of variant calls, and the detection of the homozygous (log2 ratio <−3.0) *PARD3* deletion in the aCGH data for all three samples, these variant calls likely reflect allele differences in the cultured cell line compared to the primary tissue. The correlation of the variants/mutations with aCGH data provided further description of the landscape of this PDA genome. For example homozygous *TP53* mutation occurs with loss of chromosome 17p, while a heterozygous *KRAS* mutation occurred in the background of low level chromosome 12p copy number increase, (Figure 6, Figure S11).

## Discussion

The low fragment sizes of DNA and tissue admixtures make it difficult to fully exploit FFPE samples. Increased inputs of DNA extracted from FFPE samples have been used to compensate for poor quality templates in labeling and hybridization steps. For example a minimum of 2 µg of DNA from bulk tumor samples can provide sufficient labeled template for aCGH experiments [31], [32]. In addition, the need for high tumor content requires that samples are selected and prepared based on gross morphology assessment such as H&E staining [7]. This greatly limits the use of clinical FFPE biopsies for high definition genomics of solid tumors due to complex genomes and heterogeneous cellularity. For example, in PDA a highly lethal tumor type characterized by multiple genomic aberrations, cancer cells represent on average only 25% of the cells within the tumor [33].

Flow cytometry-based cell sorters can select, objectively measure and sort individual particles such as cells or nuclei using desired features objectively defined by fluorescent and light scattering parameters in a flow stream. Recent advances in this technology provide high throughput flow rates and the detection of relatively rare events in dilute admixed samples, enabling the application of DNA content based flow cytometry assays for high definition analyses of human cancer biopsies [34]. Our flow sorting assays provide intact nuclei for DNA extraction, eliminate the need and bias to preselect samples based on tumor content and non-quantitative morphology measures, and greatly increase the number of samples that can be used for analyses. The sorting efficiencies of FF and FFPE samples can be significantly affected by the presence of debris, aggregates, and sliced nuclei. To maintain sorting efficiencies at relatively high levels (>80%) and high yields and purities of sorted samples the differential pressure of the core and the sheath fluids can be increased but cannot be >1. Slow sort rates while maintaining optimal differential pressure of flow stream improves efficiency of sorts and the overall yield of intact nuclei. However the greatest variable in our sorting was the origin of the tissue. For example TNBC sorted more efficiently than did PDA samples for both FF and FFPE samples.

Gating based on DNA content provides a robust quantitative measure for identifying and sorting tumor populations from samples of interest. For example the 3.0N population sorted from a FF PDA sample was detected 3 years later in an FFPE sample from the same tissue (Figure 5). The ploidy and the relative distribution of each population present in a biopsy can be recovered by fitting the G_0_/G_1_ and G_2_/M peaks as Gaussian curves and the S phase distribution as a Gaussian broadening distribution. The DNA content histograms from tumor tissue are frequently suboptimal (broad c.v’s, high debris and aggregation) and often complex (multiple overlapping peaks and cell cycles) with frequent skewing and non-Gaussian peak shapes. This is even truer for FFPE specimens that often contain higher levels of damaged or fragmented nuclei (debris) resulting in events usually most visible to the left of the diploid G_1_ peak and that fall rapidly to baseline (Figures S4,S9). For reproducible phase measurements we typically acquire 10,000 events. However if a substantial proportion of events are from debris or aggregates, the total number of events acquired must be correspondingly higher in order to assure the required minimum number of intact single nuclei for accurate curve fitting.

Different reports have shown that tumor cells can be efficiently sorted from FFPE samples with DNA content based assays and used for genomic analysis [35], [36]. These studies have typically relied on PCR based assays including SNP arrays. These assays have limited resolution based on the ability to distinguish homozygous from partial copy number losses, the mapping of breakpoints and focal amplicons, and in the number of genes and loci interrogated. Furthermore SNP arrays typically require the preparation of platform-specific reduced complexity samples for optimal results limiting the utility of DNA prepared from each sorted sample. In contrast our methods use whole genome templates that are compatible with a wide variety of high definition assays including aCGH and NGS. For aCGH analysis, short DNAse 1 digestion of genomic DNAs extracted directly from sorted nuclei or with amplified DNAs from FF or FFPE samples provides uniform templates for labeling [32]. The resolution of our assays with purified sorted samples enables discrimination of single copy loss from homozygous loss and the mapping of amplicon and deletion boundaries in each tumor genome.

Current inputs for NGS libraries are typically 1–3 µg of genomic DNA. Our flow assays can efficiently sort sufficient numbers of nuclei to provide those inputs. However sample availability, the quality of the FFPE preparation, and the cellular heterogeneity of the tumor frequently limit the number of samples that can be analyzed. Our direct comparison of aCGH data using template prepared from cell line genomic DNA,phi29 amplified FF DNA, and SPIA-amplified DNA from sorted FFPE samples validates the linearity of this amplification method (Figure 4, Figures S9, S10). Our subsequent analysis of sorted FFPE samples for NGS exploited a PDA cell line whose exome has been extensively studied as a control with known somatic mutations. The primary FF tissue from which the cell line was derived and the corresponding FFPE blocks provided a unique sample set for validating our sorting-based analyses. The overlap of unique reads and the detection of known mutations across the 3 independent sample preparations demonstrate that sorted FFPE samples can be used for NGS. Thus, the linear whole genome amplification of sorted FFPE samples is an efficient method to extend both aCGH and NGS to these highly informative clinical tissues.

In contrast to the cell line and the matching 3.0N population the total diploid sorted fractions from the PDA tissues were non-aberrant by aCGH analysis. However a low (<5–10%) number of reads for some mutations present in the aneuploid fraction (e.g. *KRAS*) were observed in the NGS data for the total diploid fraction in both the amplified and unamplified samples (Figure S12). The total diploid peaks in DNA content based flow sorted tumor samples may contain admixtures of neoplastic and non-neoplastic cell types. To determine whether these low frequency mutation reads represent subpopulations of neoplastic cells we used a DAPI/cytokeratin 19 and a DAPI/vimentin flow assay to resort the biopsy. The cytokeratin 19^+^ and the vimentin^+^ diploid populations each had the heterozygous *KRAS* mutation detected of the aneuploid population and cell line (Figures S13, S14). However, only the small (5–10%) cytokeratin 19^+^ diploid population had the clonal homozygous *TP53* mutation and an aCGH profile that matched the 3.0N population and the cell line. Thus the 2N cytokeratin^+^ population represents a co-existing population with a diploid by flow cytometry DNA content. In contrast the diploid *KRAS* ^mut^, *TP53* ^wt^ population was normal by aCGH and represents a third clonal population in this biopsy that is either from an earlier stage of disease or is a non-progressing neoplastic population. Our ability to resort this tissue provides a unique approach to validate our NGS results and confirm the presence of distinct clonal populations. We propose that this iterative approach can exploit the detection of low frequency reads in NGS data to provide even deeper clonal analysis.

Recent studies have used increased numbers (deep) of sequencing reads to assess the presence of multiple tumor populations in samples of interest [8], [37], [38]. However biopsies frequently contain multiple clonal populations of neoplastic cells that cannot be distinguished by morphology alone [27]. Thus analysis of even highly tumor cell-enriched bulk cancer samples, including those prepared by LCM, cannot accurately distinguish whether aberrations in a tumor are present in a single cancer genome or if they are distributed in multiple clonal populations in each biopsy. In contrast our highly sensitive and quantitative sorting assays provide pure objectively defined populations prior to analysis. The deep unbiased clonal profiling of sorted FF and FFPE samples provides a valuable methodology to advance the development of personalized therapies for patients with cancer.

## Supporting Information

Figure S1 Flow sorted FFPE sample input and aCGH. Sample input and aberration detection in flow sorted triple negative breast cancer (TNBC) formalin fixed paraffin embedded (FFPE) tissue PS02 01557 E3. A) Diploid (green) and aneuploid (red) peaks sorted from FFPE sample. B) Cell cycle and ploidy analysis of sorted populations. C) The >1.0 log_2_ratio gain (red arrow) at 8q23.1-q23.2 is detected in aCGH data from 10,000 (10 k), 25,000 (25 k), and 50,000 (50 k) nuclei. In contrast the <1.0 log_2_ratio gain (black arrow) gain in the same region is seen in the 25 k and 50 k data, and the <−1.0 log_2_ratio deletion (blue arrow) targeting *PKDH1L1* is only detected in the 50 k data. D) Detection of <−3.0 log_2_ ratio homozygous deletion (blue arrow) of *TNFAIP8* at 5q23.1 in 50 k data. Shaded areas denote ADM2-defined aberrant intervals.
(TIF)

Figure S2 Aberration detection and aCGH of flow sorted breast carcinoma FFPE sample. aCGH analysis of sorted diploid and aneuploid populations of triple negative breast cancer (TNBC) formalin fixed paraffin embedded (FFPE) tissue PS03 4398 B2. A) Cell cycle and ploidy analysis of sorted 2.0N and 3.1N populations. B) Whole genome plots of 2.0N and 3.1N sorted populations. C) Gene level view of focal deletions in *PARD3* and *ERBB4* genes in 3.1N genome. Shaded areas denote ADM2-defined aberrant intervals.
(TIF)

Figure S3 Aberration detection and aCGH of flow sorted breast carcinoma FFPE sample. aCGH analysis of sorted aneuploid populations of triple negative breast cancer (TNBC) formalin fixed paraffin embedded (FFPE) tissue SS04 4239 A2. A) Cell cycle and ploidy analysis of sorted 2.0N and 3.4N populations. B) Whole genome plots of sorted 3.4N population. C–D) Chromosome and gene level view of focal 21q21.2 amplicon that includes the *USP25* locus in 3.4N genome. Shaded areas denote ADM2-defined aberrant intervals.
(TIF)

Figure S4 Aberration detection and aCGH of flow sorted bladder carcinoma FFPE sample. aCGH analysis of sorted aneuploid population from bladder carcinoma formalin fixed paraffin embedded (FFPE) tissue B33251. Flow sorting of bladder carcinoma formalin fixed paraffin embedded (FFPE) tissue B33251. A) Cell cycle and ploidy analysis of sorted 2.0N and 3.1N populations. B) Whole genome plots of sorted 3.1N population. C–D) Chromosome 11 and gene level view of focal 11q13.3 amplicon that includes the *CCND1* locus in 3.1N genome. Shaded areas denote ADM2-defined aberrant intervals.
(TIF)

Figure S5 Aberration detection and aCGH of flow sorted glioblastoma FFPE sample. aCGH analysis of sorted aneuploid population from glioblastoma multiforme formalin fixed paraffin embedded (FFPE) tissue. A) Whole genome plots of sorted tumor population. B–C) Chromosome 7 and gene level view of focal 7p11 amplicon that includes the *EGFR* locus. Shaded areas denote ADM2-defined aberrant intervals.
(TIF)

Figure S6 Aberration detection and aCGH of flow sorted ovarian carcinoma FFPE sample. Flow sorting and aCGH analysis of small cell carcinoma of the ovary (SCCO) formalin fixed paraffin embedded (FFPE) tissue 006. A) Diploid (blue) and tetraploid (red) populations sorted from the FFPE sample. B–C) Whole genome and chromosome 1 aCGH plots of 4.0N genome. D) Gene view of 1p36.22 and mapping of breakpoint at *CASZ1* locus. Shaded areas denote ADM2-defined aberrant intervals.
(TIF)

Figure S7 FISH validation of genomic aberrations detected by array CGH. A and B) FISH hybridization on the pancreatic adenocarcinoma B3733 (A) reveals a homozygous *CDKN2A* gene deletion, whereas the control pancreas tissue (B) harbors two intact copies of the genes *CDKN2A* (green) and of the centromere 9 (red). Red and green arrows pointing towards centromere 9 and *CDKN2A* gene signals, respectively. C) FISH hybridization with the Cyclin D1 FISH probe on the bladder carcinoma B33251 shows genomic amplification of the *CCND1* gene (green arrow). Red and green arrows pointing towards centromere 11 and *CCND1* gene signals, respectively.
(TIF)

Figure S8 Whole genome amplification of sorted FFPE samples. Summary of aCGH quality control (Q.C.) metrics for flow sorted pancreatic ductal adenocarcinoma (PDA) FFPE samples using SPIA amplified DNA from 10,000, 25,000, and 50,000 nuclei as input. A) PDA sample 120-02. B) PDA sample 3733.
(TIF)

Figure S9 Use of amplified sorted FFPE samples for aCGH. Comparison of aCGH results using amplified and non-amplified DNA from flow sorted pancreatic ductal adenocarcinoma (PDA) formalin fixed paraffin embedded (FFPE) tissue 120-02. The DNA extracted from 50,000 sorted aneuploid (3.4N) nuclei was amplified using the SPIA method prior to labeling and hybridization to 400 k CGH arrays. A) Diploid (blue) and aneuploid (red) populations sorted from the FFPE sample in the presence of extensive debris in tissue sample. B) Whole genome aCGH plot of 3.4N genome. C-D) Chromosome 9 and *CDKN2A* results using non-amplified DNA and SPIA amplified DNA from the sorted 3.4N population. Shaded areas denote ADM2-defined aberrant intervals.
(TIF)

Figure S10 Use of amplified sorted FFPE samples for aCGH. Comparison of breakpoint mapping in the 3.4N population flow sorted from fresh frozen (FF) and formalin fixed paraffin embedded (FFPE) pancreatic ductal adenocarcinoma (PDA) tissues from sample 120-2. aCGH analysis was done in the 3.4N population using sorted phi29 amplified DNA from sorted FF (blue line), and unamplified (orange line) and SPIA-amplified (red line) DNA from sorted FFPE. CGH gene view plots for A) 9p21.3. B) 20p11.23. C) 15q21.1. D) 8q24.12. Shaded areas denote ADM2-defined aberrant intervals.
(TIF)

Figure S11 Use of amplified sorted FFPE samples for whole exome sequencing. Whole exome sequencing of sorted fresh frozen (FF) and formalin fixed paraffin embedded (FFPE) pancreatic ductal adenocarcinoma (PDA) tissue. A) Flow sorted 3.0N tumor population from PDA tissue. B) Homozygous *CTNNA3* mutation in sorted FF and FFPE tissues, and matching cell line. C) Chromosome 10 aCGH plot of 3.0N population from sorted FF sample. D) Heterozygous *VWF* mutation in sorted FF and FFPE tissues, and matching cell line. E) Chromosome 12 aCGH plot of 3.0N population from sorted FF sample.
(TIF)

Figure S12 Use of amplified sorted FFPE samples for whole exome sequencing. Whole exome sequencing of aneuploid and diploid population in pancreatic ductal adenocarcinoma (PDA) tissue. A) Heterozygous *KRAS* mutation detected in flow sorted 3.0N tumor population. B) Homozygous *TP53* mutation detected inflow sorted 3.0N tumor population, and absent in the flow sorted 2.0N population from PDA tissue.
(TIF)

Figure S13 Detection and multiparameter sorting of diploid subpopulations. A) Multiparameter DAPI/cytokeratin 19 sorting of pancreatic ductal adenocarcinoma (PDA) biopsy. A) Scatter plot (left) and histogram (right) of diploid and aneuploid populations. B) Multiparameter DAPI/vimentin sorting of PDA biopsy. Scatter plot (left) and histogram (right) of diploid and aneuploid populations.
(TIF)

Figure S14 Genomic analysis of multiparameter sorted pancreatic ductal adenocarcinoma (PDA) populations. A) Targeted resequencing of *KRAS*. Heterozygous mutation detected in cell line (top), sorted cytokeratin^+^ diploid (middle), and sorted vimentin^+^ diploid (bottom) PDA samples. B) Targeted resequencing of *TP53*. Homozygous *TP53* mutation detected in cell line (top), sorted cytokeratin+ diploid (middle), but absent in sorted vimentin^+^ diploid (bottom) PDA. C) Chromosome 10 p and *PARD3* locus CGH analysis of flow sorted 3.0N aneuploid, total diploid (2.0N), cytokeratin 19^+^2.0N, and vimentin^+^2.0N populations. Shaded areas denote ADM2-defined aberrant intervals.
(TIF)

Table S1 Summary statistics and hybrid selection metrics (HsMetrics). Results were reported by Picard tool for exome alignment data for sorted FF (A10-46), sorted FFPE (A10-AT), and matching cell line (A10-74).
(PDF)

Table S2 Exome alignment summary metrics. Results reported by Picard for sorted FF (A10-46), sorted FFPE (A10-AT), and matching cell line (A10-74).
(PDF)
