# Supplementary figures and images for: Deep Clonal Profiling of Formalin Fixed Paraffin Embedded Clinical Samples
Source: PLoS One. 2012 Nov 30;7(11):e50586. doi: 10.1371/journal.pone.0050586 (PMC3511535; doi:10.1371/journal.pone.0050586)

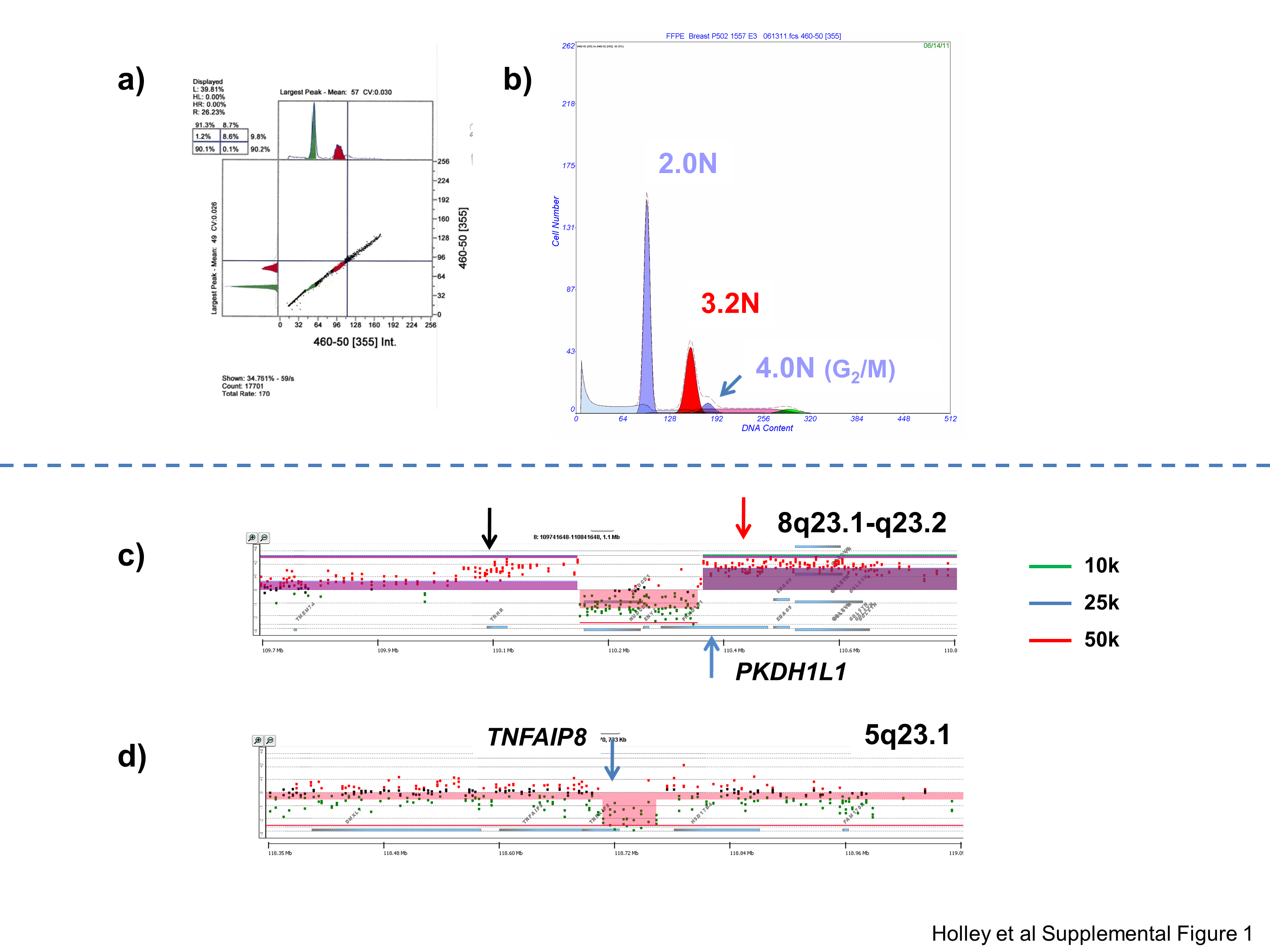

Supplement: Figure S1 — Flow sorted FFPE sample input and aCGH. Sample input and aberration detection in flow sorted triple negative breast cancer (TNBC) formalin fixed paraffin embedded (FFPE) tissue PS02 01557 E3. A) Diploid (green) and aneuploid (red) peaks sorted from FFPE sample. B) Cell cycle and ploidy analysis of sorted populations. C) The >1.0 log2ratio gain (red arrow) at 8q23.1-q23.2 is detected in aCGH data from 10,000 (10 k), 25,000 (25 k), and 50,000 (50 k) nuclei. In contrast the <1.0 log2ratio gain (black arrow) gain in the same region is seen in the 25 k and 50 k data, and the <−1.0 log2ratio deletion (blue arrow) targeting PKDH1L1 is only detected in the 50 k data. D) Detection of <−3.0 log2 ratio homozygous deletion (blue arrow) of TNFAIP8 at 5q23.1 in 50 k data. Shaded areas denote ADM2-defined aberrant intervals. (TIF) [file pone.0050586.s001.tif]

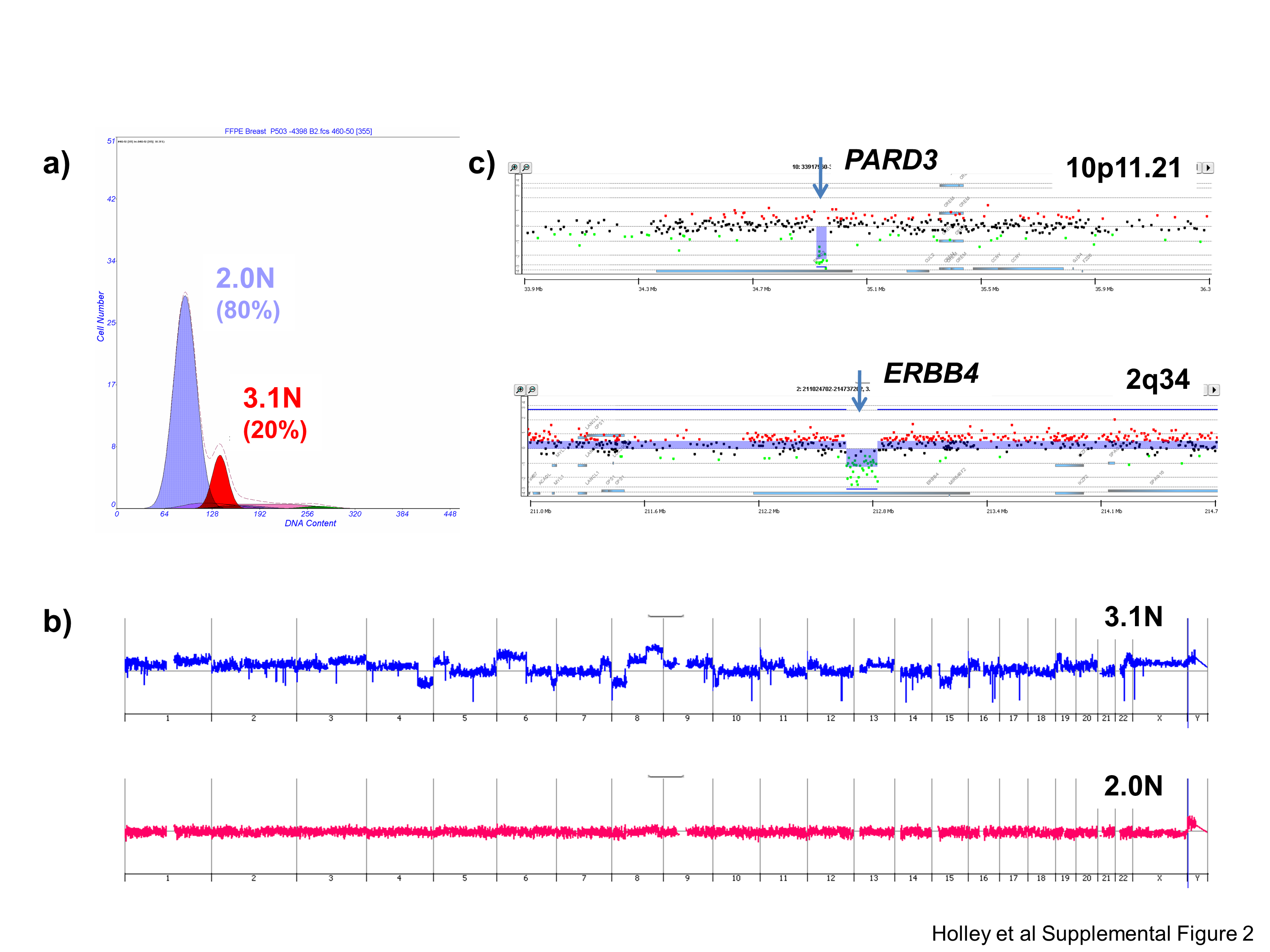

Supplement: Figure S2 — Aberration detection and aCGH of flow sorted breast carcinoma FFPE sample. aCGH analysis of sorted diploid and aneuploid populations of triple negative breast cancer (TNBC) formalin fixed paraffin embedded (FFPE) tissue PS03 4398 B2. A) Cell cycle and ploidy analysis of sorted 2.0N and 3.1N populations. B) Whole genome plots of 2.0N and 3.1N sorted populations. C) Gene level view of focal deletions in PARD3 and ERBB4 genes in 3.1N genome. Shaded areas denote ADM2-defined aberrant intervals. (TIF) [file pone.0050586.s002.tif]

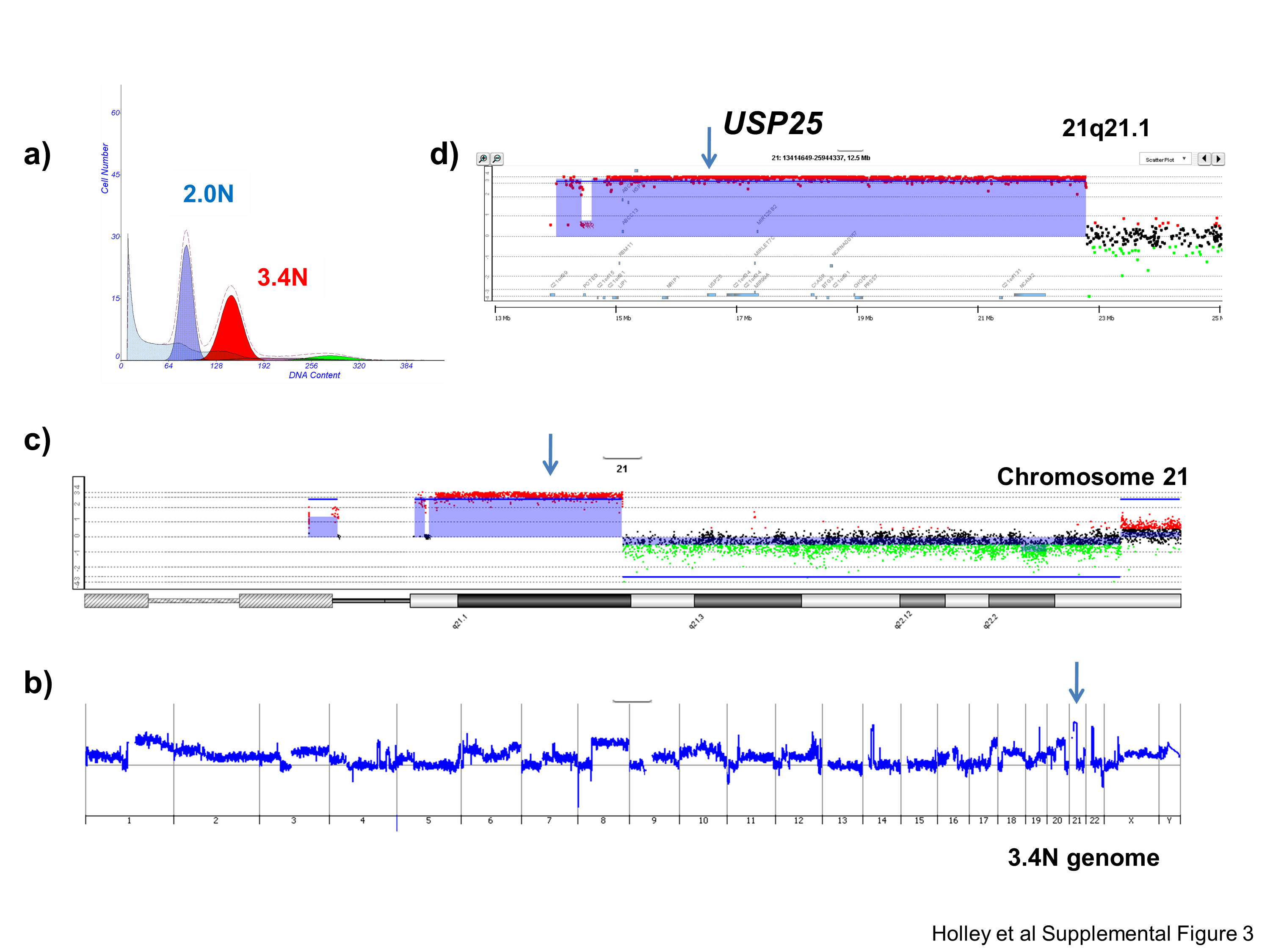

Supplement: Figure S3 — Aberration detection and aCGH of flow sorted breast carcinoma FFPE sample. aCGH analysis of sorted aneuploid populations of triple negative breast cancer (TNBC) formalin fixed paraffin embedded (FFPE) tissue SS04 4239 A2. A) Cell cycle and ploidy analysis of sorted 2.0N and 3.4N populations. B) Whole genome plots of sorted 3.4N population. C–D) Chromosome and gene level view of focal 21q21.2 amplicon that includes the USP25 locus in 3.4N genome. Shaded areas denote ADM2-defined aberrant intervals. (TIF) [file pone.0050586.s003.tif]

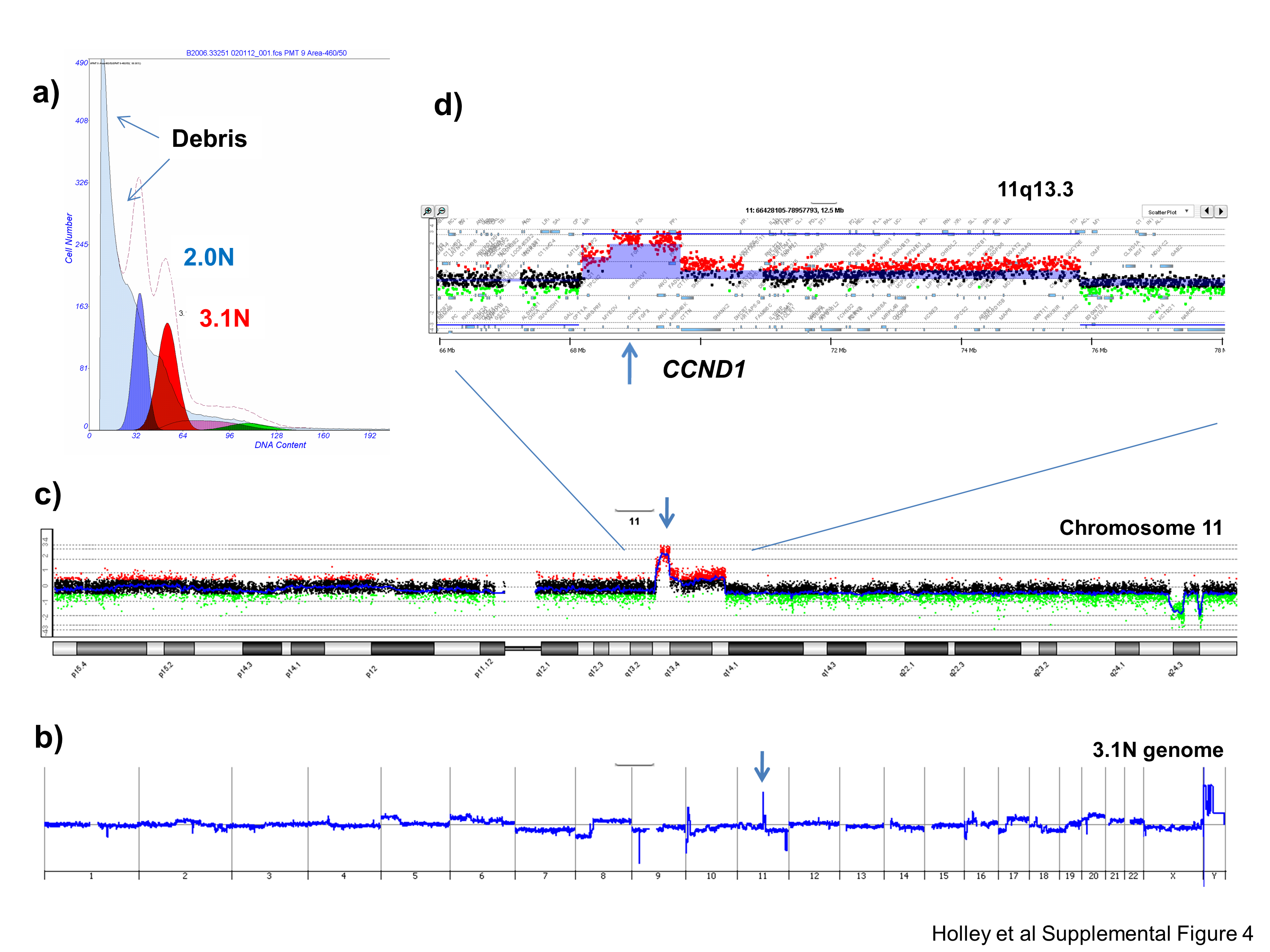

Supplement: Figure S4 — Aberration detection and aCGH of flow sorted bladder carcinoma FFPE sample. aCGH analysis of sorted aneuploid population from bladder carcinoma formalin fixed paraffin embedded (FFPE) tissue B33251. Flow sorting of bladder carcinoma formalin fixed paraffin embedded (FFPE) tissue B33251. A) Cell cycle and ploidy analysis of sorted 2.0N and 3.1N populations. B) Whole genome plots of sorted 3.1N population. C–D) Chromosome 11 and gene level view of focal 11q13.3 amplicon that includes the CCND1 locus in 3.1N genome. Shaded areas denote ADM2-defined aberrant intervals. (TIF) [file pone.0050586.s004.tif]

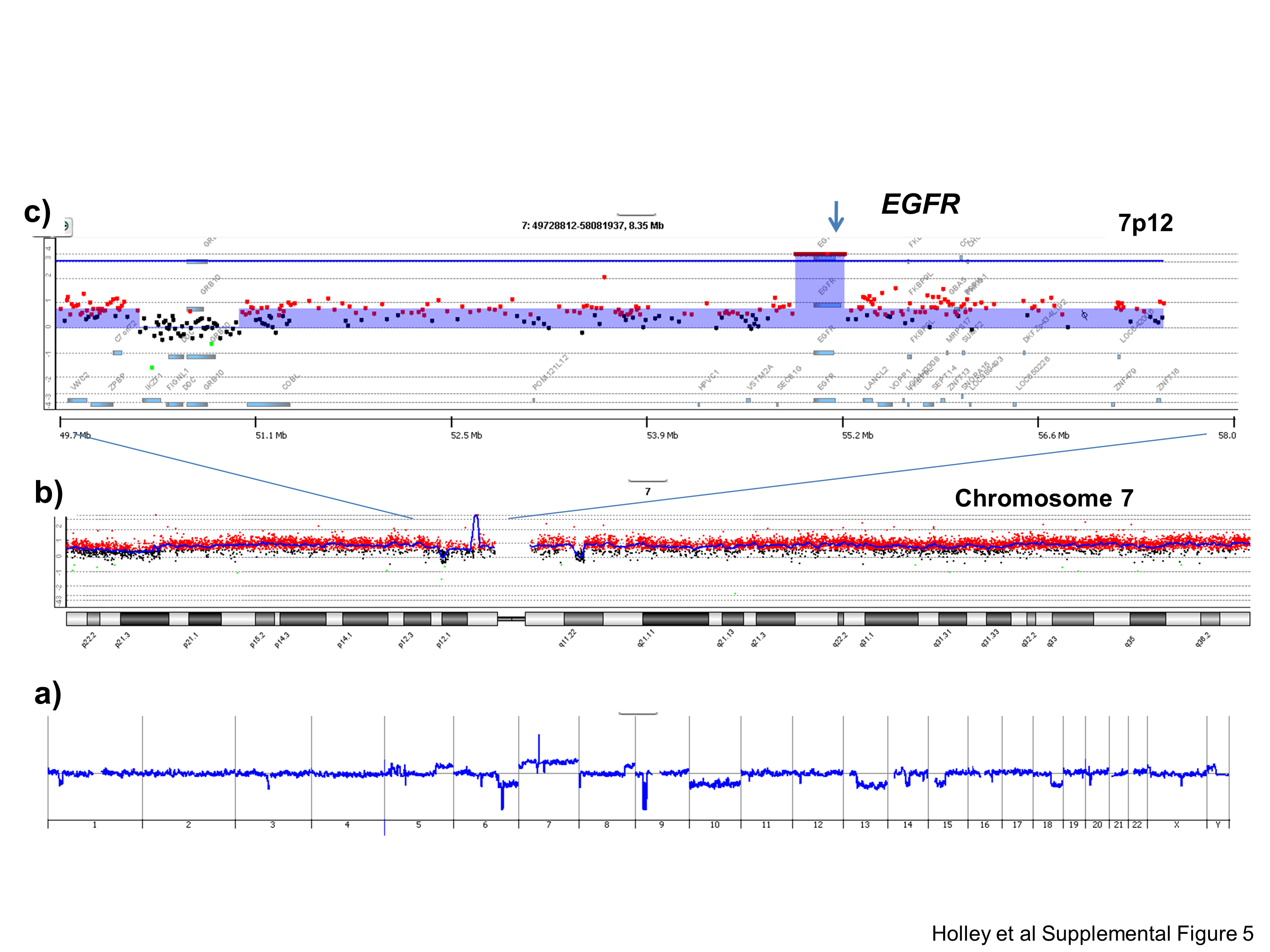

Supplement: Figure S5 — Aberration detection and aCGH of flow sorted glioblastoma FFPE sample. aCGH analysis of sorted aneuploid population from glioblastoma multiforme formalin fixed paraffin embedded (FFPE) tissue. A) Whole genome plots of sorted tumor population. B–C) Chromosome 7 and gene level view of focal 7p11 amplicon that includes the EGFR locus. Shaded areas denote ADM2-defined aberrant intervals. (TIF) [file pone.0050586.s005.tif]

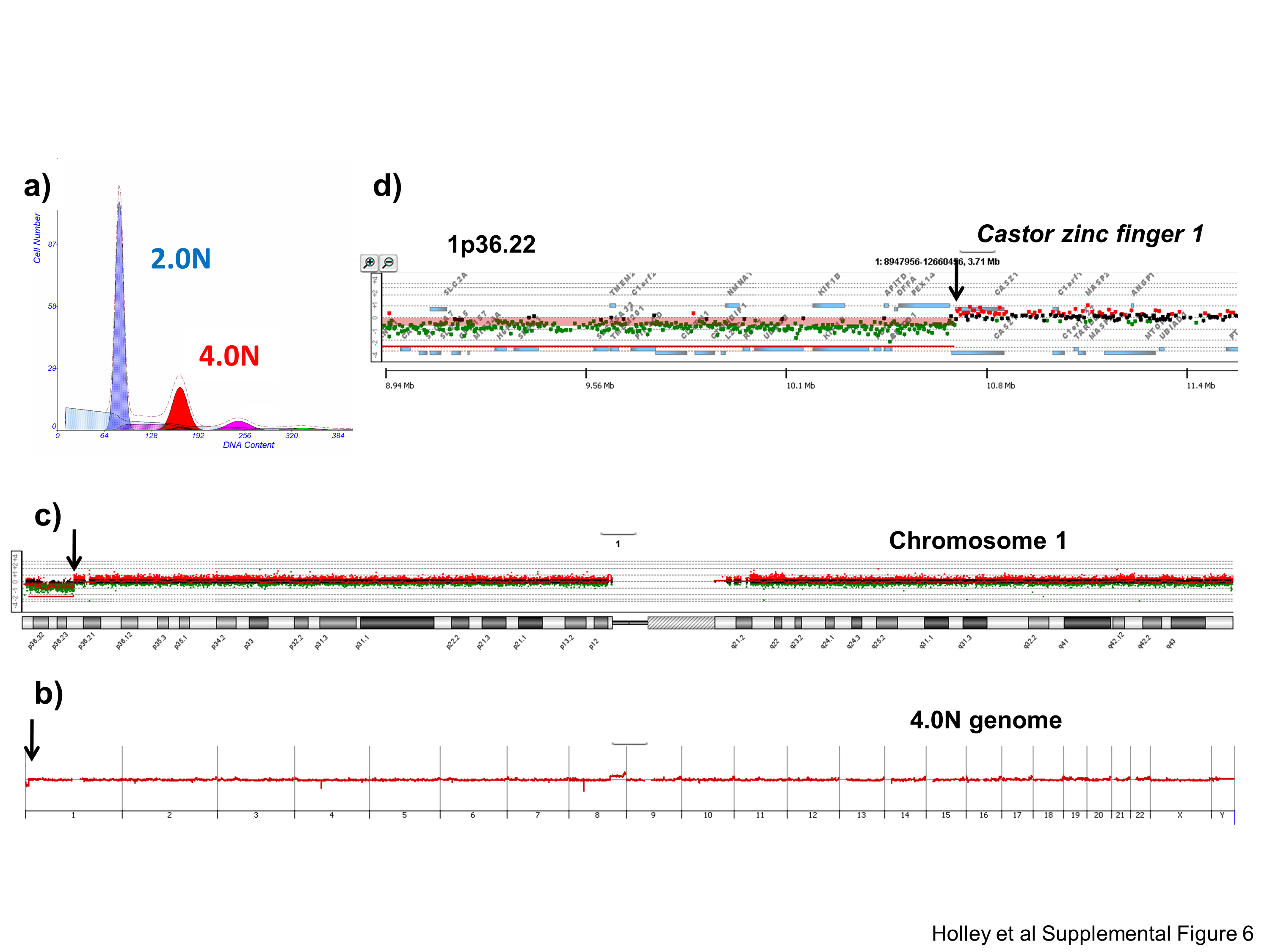

Supplement: Figure S6 — Aberration detection and aCGH of flow sorted ovarian carcinoma FFPE sample. Flow sorting and aCGH analysis of small cell carcinoma of the ovary (SCCO) formalin fixed paraffin embedded (FFPE) tissue 006. A) Diploid (blue) and tetraploid (red) populations sorted from the FFPE sample. B–C) Whole genome and chromosome 1 aCGH plots of 4.0N genome. D) Gene view of 1p36.22 and mapping of breakpoint at CASZ1 locus. Shaded areas denote ADM2-defined aberrant intervals. (TIF) [file pone.0050586.s006.tif]

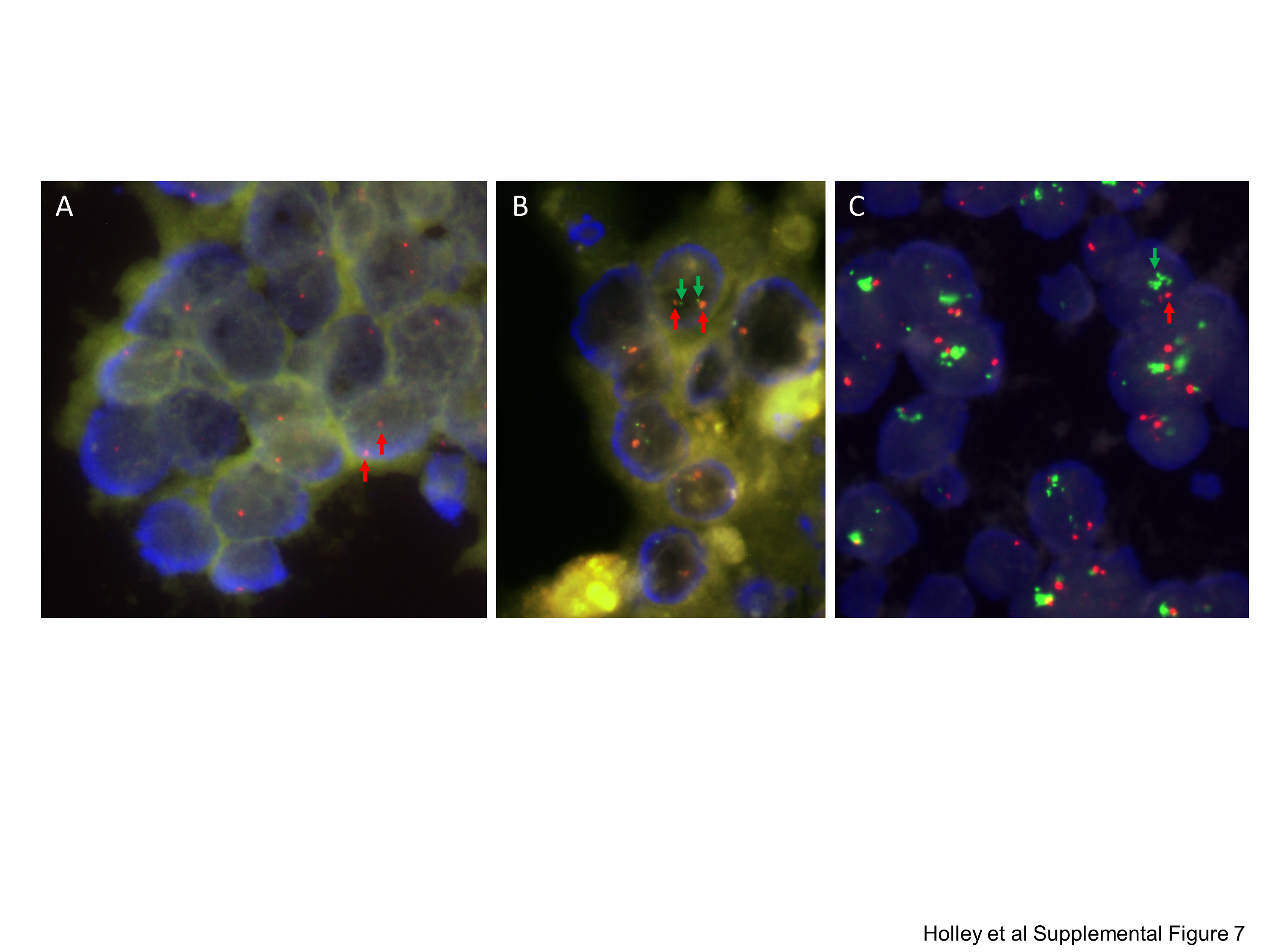

Supplement: Figure S7 — FISH validation of genomic aberrations detected by array CGH. A and B) FISH hybridization on the pancreatic adenocarcinoma B3733 (A) reveals a homozygous CDKN2A gene deletion, whereas the control pancreas tissue (B) harbors two intact copies of the genes CDKN2A (green) and of the centromere 9 (red). Red and green arrows pointing towards centromere 9 and CDKN2A gene signals, respectively. C) FISH hybridization with the Cyclin D1 FISH probe on the bladder carcinoma B33251 shows genomic amplification of the CCND1 gene (green arrow). Red and green arrows pointing towards centromere 11 and CCND1 gene signals, respectively. (TIF) [file pone.0050586.s007.tif]

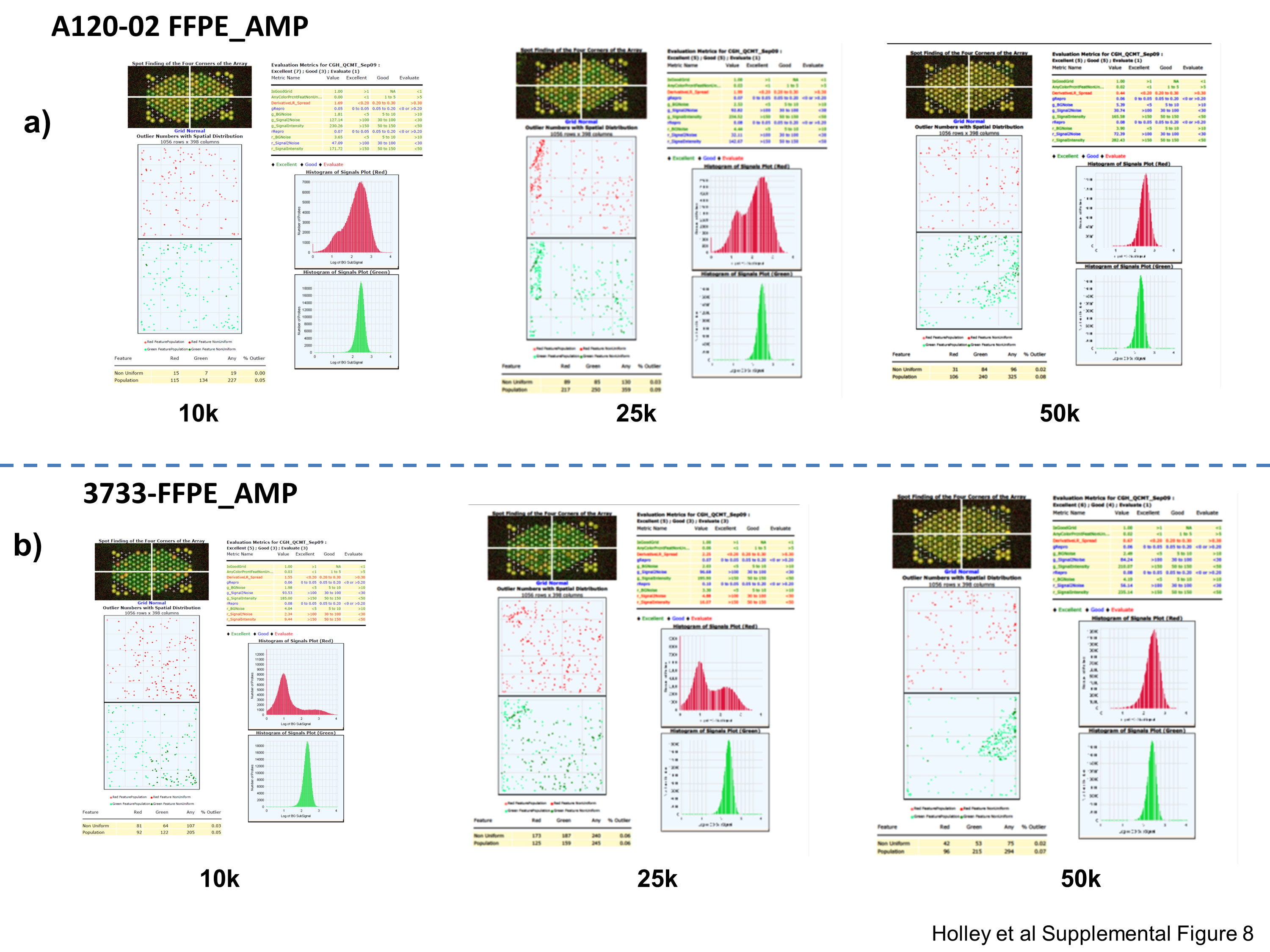

Supplement: Figure S8 — Whole genome amplification of sorted FFPE samples. Summary of aCGH quality control (Q.C.) metrics for flow sorted pancreatic ductal adenocarcinoma (PDA) FFPE samples using SPIA amplified DNA from 10,000, 25,000, and 50,000 nuclei as input. A) PDA sample 120-02. B) PDA sample 3733. (TIF) [file pone.0050586.s008.tif]

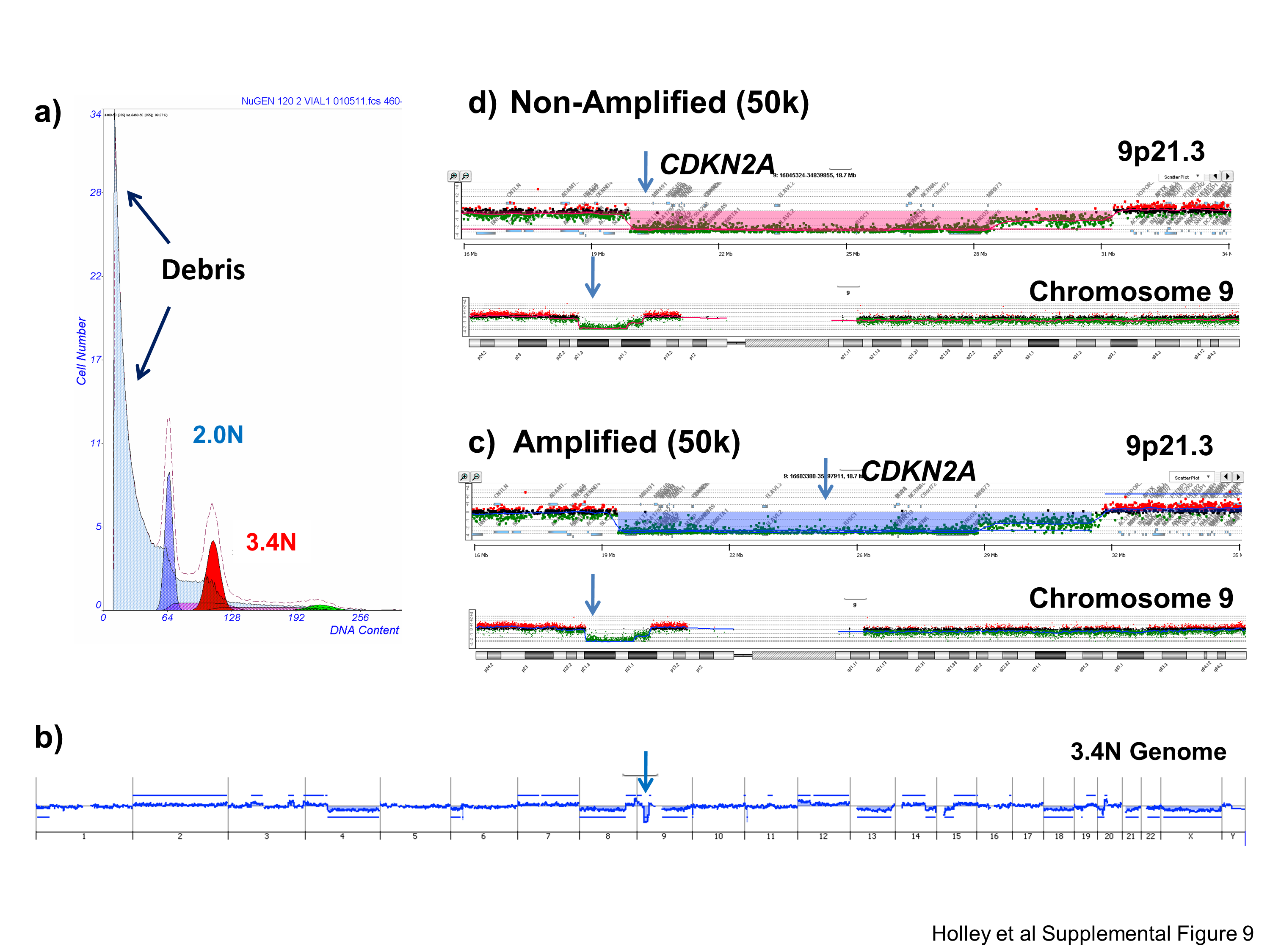

Supplement: Figure S9 — Use of amplified sorted FFPE samples for aCGH. Comparison of aCGH results using amplified and non-amplified DNA from flow sorted pancreatic ductal adenocarcinoma (PDA) formalin fixed paraffin embedded (FFPE) tissue 120-02. The DNA extracted from 50,000 sorted aneuploid (3.4N) nuclei was amplified using the SPIA method prior to labeling and hybridization to 400 k CGH arrays. A) Diploid (blue) and aneuploid (red) populations sorted from the FFPE sample in the presence of extensive debris in tissue sample. B) Whole genome aCGH plot of 3.4N genome. C-D) Chromosome 9 and CDKN2A results using non-amplified DNA and SPIA amplified DNA from the sorted 3.4N population. Shaded areas denote ADM2-defined aberrant intervals. (TIF) [file pone.0050586.s009.tif]

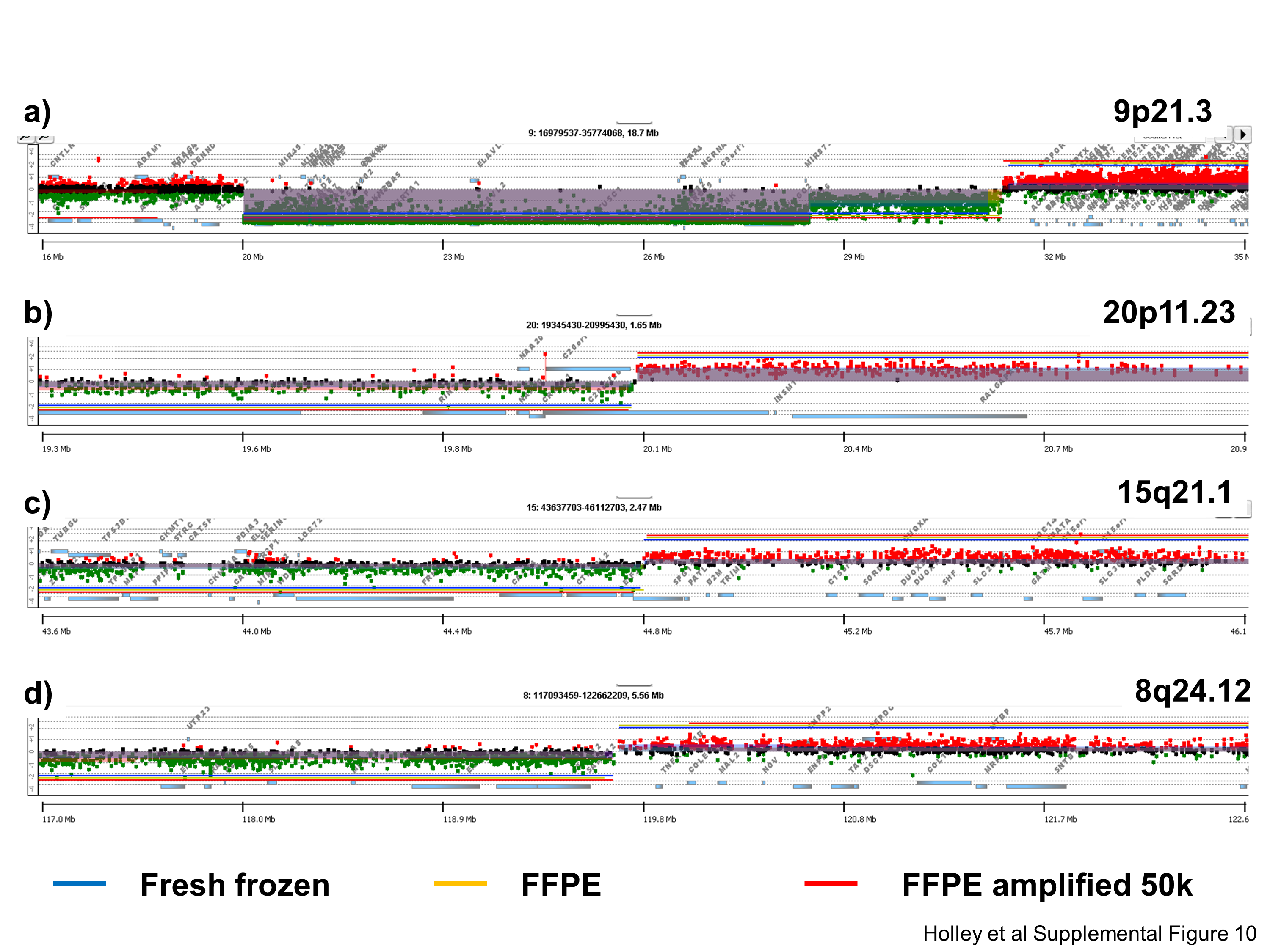

Supplement: Figure S10 — Use of amplified sorted FFPE samples for aCGH. Comparison of breakpoint mapping in the 3.4N population flow sorted from fresh frozen (FF) and formalin fixed paraffin embedded (FFPE) pancreatic ductal adenocarcinoma (PDA) tissues from sample 120-2. aCGH analysis was done in the 3.4N population using sorted phi29 amplified DNA from sorted FF (blue line), and unamplified (orange line) and SPIA-amplified (red line) DNA from sorted FFPE. CGH gene view plots for A) 9p21.3. B) 20p11.23. C) 15q21.1. D) 8q24.12. Shaded areas denote ADM2-defined aberrant intervals. (TIF) [file pone.0050586.s010.tif]

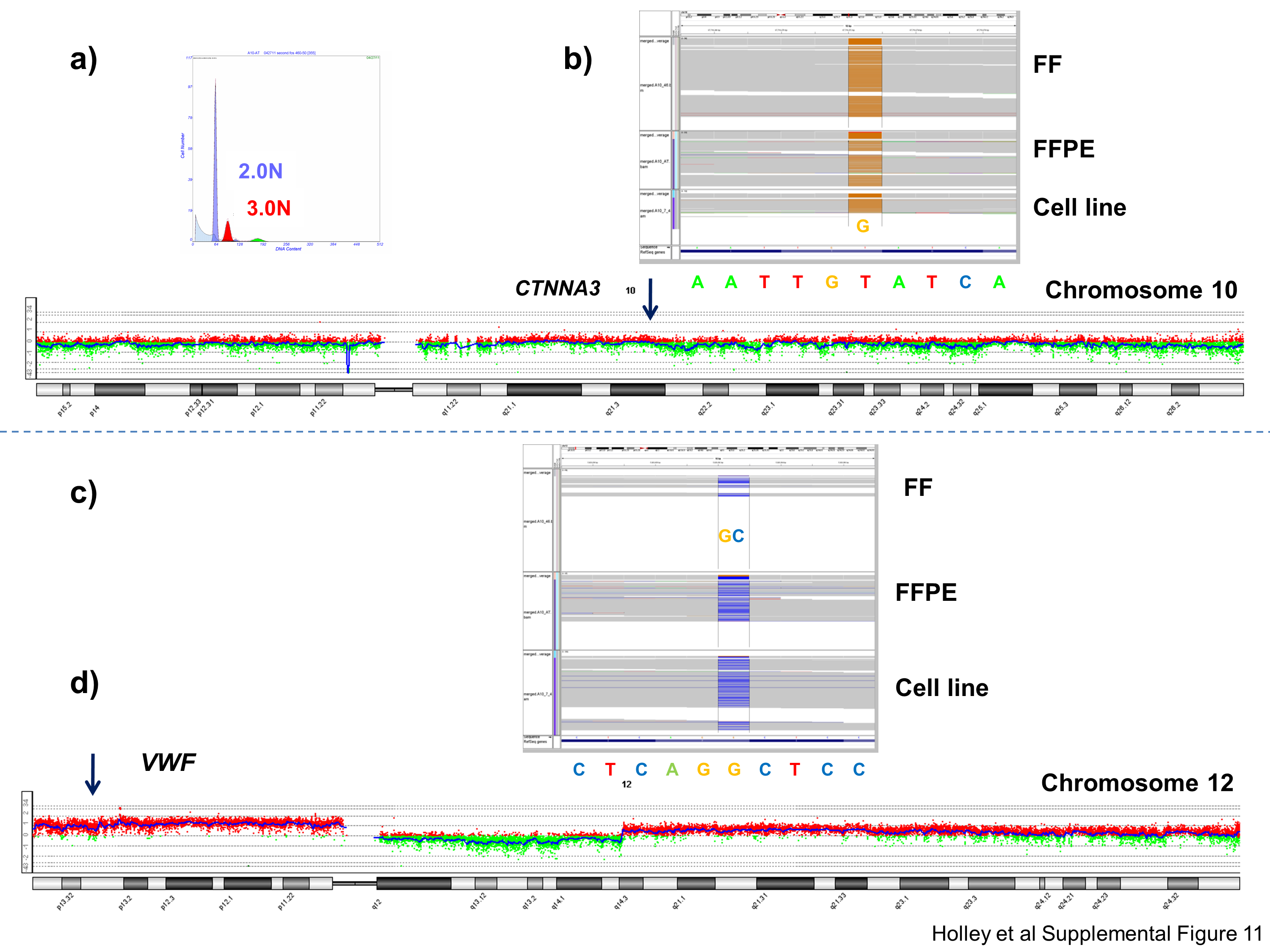

Supplement: Figure S11 — Use of amplified sorted FFPE samples for whole exome sequencing. Whole exome sequencing of sorted fresh frozen (FF) and formalin fixed paraffin embedded (FFPE) pancreatic ductal adenocarcinoma (PDA) tissue. A) Flow sorted 3.0N tumor population from PDA tissue. B) Homozygous CTNNA3 mutation in sorted FF and FFPE tissues, and matching cell line. C) Chromosome 10 aCGH plot of 3.0N population from sorted FF sample. D) Heterozygous VWF mutation in sorted FF and FFPE tissues, and matching cell line. E) Chromosome 12 aCGH plot of 3.0N population from sorted FF sample. (TIF) [file pone.0050586.s011.tif]

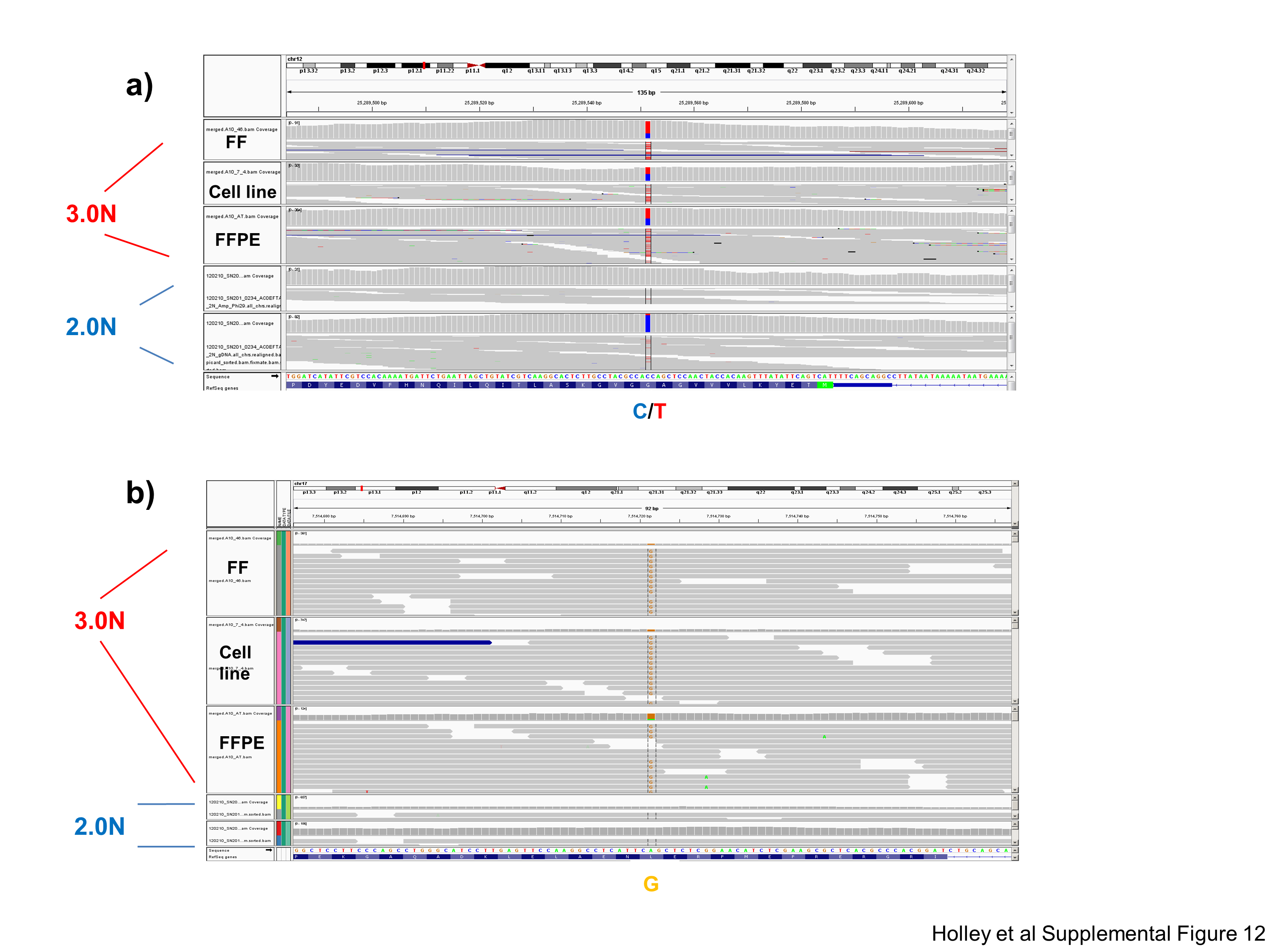

Supplement: Figure S12 — Use of amplified sorted FFPE samples for whole exome sequencing. Whole exome sequencing of aneuploid and diploid population in pancreatic ductal adenocarcinoma (PDA) tissue. A) Heterozygous KRAS mutation detected in flow sorted 3.0N tumor population. B) Homozygous TP53 mutation detected inflow sorted 3.0N tumor population, and absent in the flow sorted 2.0N population from PDA tissue. (TIF) [file pone.0050586.s012.tif]

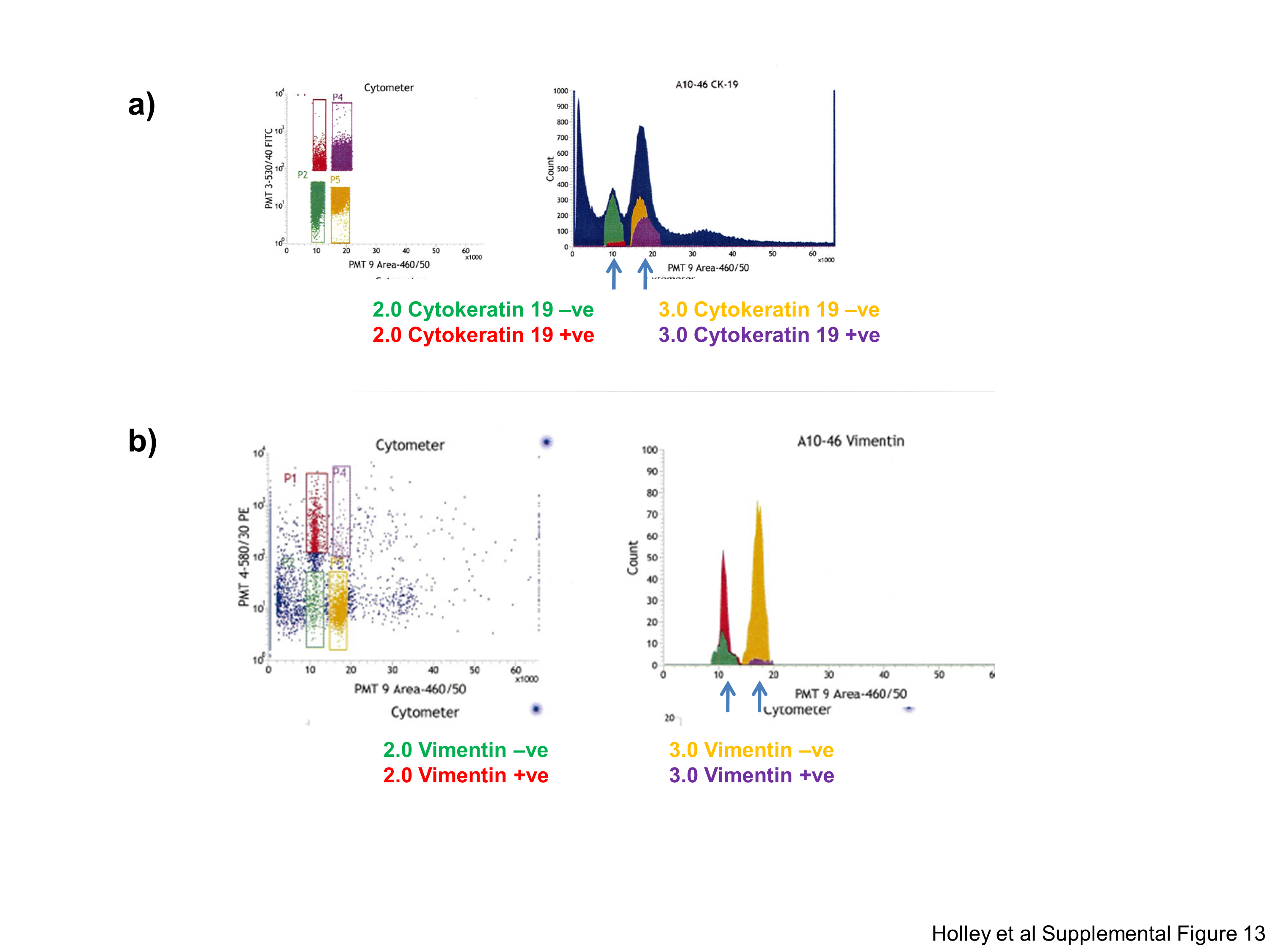

Supplement: Figure S13 — Detection and multiparameter sorting of diploid subpopulations. A) Multiparameter DAPI/cytokeratin 19 sorting of pancreatic ductal adenocarcinoma (PDA) biopsy. A) Scatter plot (left) and histogram (right) of diploid and aneuploid populations. B) Multiparameter DAPI/vimentin sorting of PDA biopsy. Scatter plot (left) and histogram (right) of diploid and aneuploid populations. (TIF) [file pone.0050586.s013.tif]

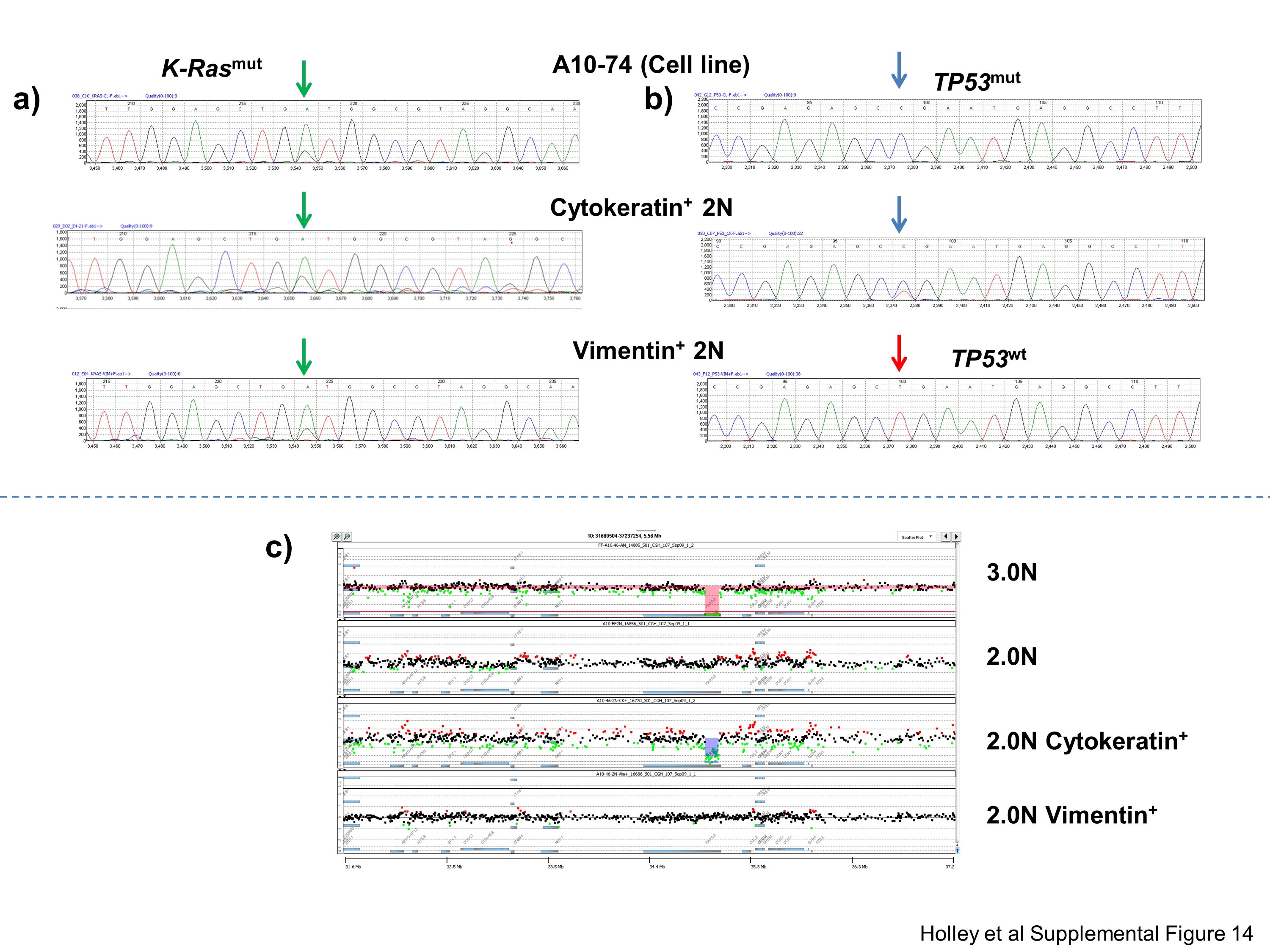

Supplement: Figure S14 — Genomic analysis of multiparameter sorted pancreatic ductal adenocarcinoma (PDA) populations. A) Targeted resequencing of KRAS. Heterozygous mutation detected in cell line (top), sorted cytokeratin+ diploid (middle), and sorted vimentin+ diploid (bottom) PDA samples. B) Targeted resequencing of TP53. Homozygous TP53 mutation detected in cell line (top), sorted cytokeratin+ diploid (middle), but absent in sorted vimentin+ diploid (bottom) PDA. C) Chromosome 10 p and PARD3 locus CGH analysis of flow sorted 3.0N aneuploid, total diploid (2.0N), cytokeratin 19+2.0N, and vimentin+2.0N populations. Shaded areas denote ADM2-defined aberrant intervals. (TIF) [file pone.0050586.s014.tif]
