# Supplementary material for: Deep Clonal Profiling of Formalin Fixed Paraffin Embedded Clinical Samples
Source: PLoS One. 2012 Nov 30;7(11):e50586. doi: 10.1371/journal.pone.0050586 (PMC3511535; doi:10.1371/journal.pone.0050586)

chr	start	end	ref	ref_reads	alt_reads	% variant	alt_allele	ref_reads	alt_reads	% variant	alt_allele	ref_reads	alt_reads	% variant	alt_allele	
chr3	1393744	1393745	G	63	189	75%	A	0	59	100%	A	60	109	64.50%	A	Match. Not called homozygous in all
chr3	115332813	115332814	G	69	247	78.16%	A	1	404	99.75%	A	15	50	76.92%	A	Match. Not called homozygous in all
chr5	24523719	24523720	T	683	46	11%	A	330	64	17%	A	129	23	15%	A	10-16% alternate allele
chr6	27899908	27899909	G	19	0	0%	-	94	69	42.33%	C	15	0	0%	-	No variant detected in 46, AT
chr6	31653209	31653210	C	24	48	66.67%	T	118	110	48.25%	T	6	5	45%	T	Match. Low coverage in AT, but visible variant
chr6	128339561	128339562	T	37	68	64.76%	A	21	27	56.25%	A	34	81	70.43%	A	Match in all
chr8	12990384	12990385	C	128	53	29.28%	T	128	65	33.68%	T	232	52	18%	.	18 to 34% alternate allele
chr10	67710271	67710272	T	9	320	97.26%	G	0	68	100%	G	21	179	89.50%	G	Match in all
chr12	5929301	5929302	G	26	40	60.61%	C	141	161	53.31%	C	75	128	63.05%	C	Match in all
chr12	21348641	21348642	G	428	157	26.84%	A	47	30	38.96%	A	187	97	34.15%	A	Match in all
chr12	25289550	25289551	C	23	58	71.60%	T	18	18	50%	T	131	197	60.06%	T	Match in all
chr12	55842540	55842541	C	35	39	52.70%	T	135	133	49.63%	T	35	7	17%	T	Match in 46, 7_4. 17% alternate allele in AT
chr15	72802199	72802200	G	11	11	50%	A	151	158	51.13%	A	15	5	25%	.	Match in 46, 7_4. 25% alternate allele in AT
chr17	7514720	7514721	A	1	32	96.97%	G	0	110	100%	G	9	47	83.93%	G	Match in 46, 7_4. 84% alt allele in AT
chr17	41451830	41451831	G	113	91	44.61%	T	385	177	31.49%	T	87	85	49.42%	T	Match in all
chr17	62616877	62616878	G	129	95	42.41%	A	56	23	29.11%	A	201	173	46.26%	A	Match in 46, AT. In 7_4, two alternate alleles T and A
chr19	4005887	4005888	C	3	0	0%	-	20	19	48.72%	A	6	0	0%	-	Low coverage in 46 and AT. Variant called in 7_4. Jones at al., 2008 paper has insertion predicted.
chr19	11005004	11005005	C	1	12	92.31%	T	0	343	100%	T	1	20	95.24%	T	Match in all
chr19	61644428	61644429	A	365	175	32.41%	G	47	43	47.78%	G	366	150	29.07%	G	Match in all
chr20	33331244	33331245	.	75	17	18%	AA del	841	236	22%	AA del	214	51	19%	AA del	Deletion observed at low frequency in all
chr20	36302824	36302825	C	186	150	44.64%	T	657	595	47.52%	T	121	71	36.98%	T	Match in all
chr20	46677551	46677552	C	40	14	25.93%	A	157	129	45.10%	A	5	2	29%	A	Match in 7_4, variant on reverse stand reads only in 46, low coverage in AT

Low coverage
Variant instead of insertion predicted

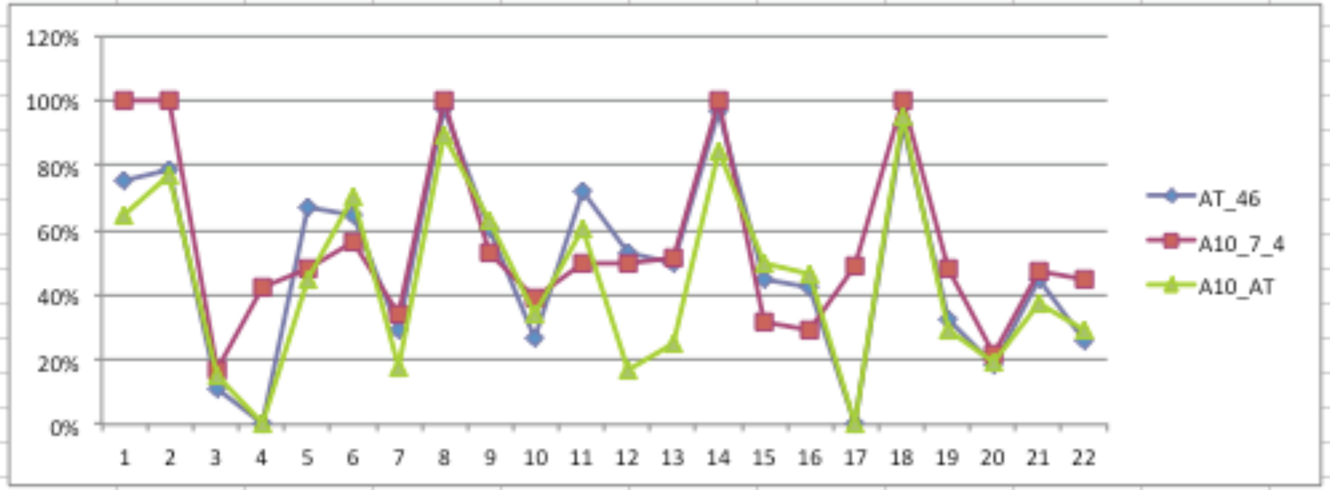

Supplement: Table S1 — Summary statistics and hybrid selection metrics (HsMetrics). Results were reported by Picard tool for exome alignment data for sorted FF (A10-46), sorted FFPE (A10-AT), and matching cell line (A10-74). (PDF) [file pone.0050586.s015.pdf]
