# Supplementary material for: Deep Clonal Profiling of Formalin Fixed Paraffin Embedded Clinical Samples
Source: PLoS One. 2012 Nov 30;7(11):e50586. doi: 10.1371/journal.pone.0050586 (PMC3511535; doi:10.1371/journal.pone.0050586)
Supplement: Table S2 — Exome alignment summary metrics. Results reported by Picard for sorted FF (A10-46), sorted FFPE (A10-AT), and matching cell line (A10-74). (PDF) [file pone.0050586.s016.pdf]

HsMetrics reported by Picard tool for exome alignment data

SAMPLE	A10_46		A10_AT		A10_7_4
READ_GROUP	C0109ABXX_A10_46_L008	110811_SN388_0267_BB0661ABXX_s3	C0109ABXX_A10_AT_L007	110811_SN388_0267_BB0661ABXX_s2	110819_SN201_207_AC02GJACXX_s5
BAIT_SET	SureSelect_All_Exon_50mb_with_annotation_hg19_bed.lft.to.hg18.bed	SureSelect_All_Exon_50mb_with_annotation_hg19_bed.lft.to.hg18.bed	SureSelect_All_Exon_50mb_with_annotation_hg19_bed.lft.to.hg18.bed	SureSelect_All_Exon_50mb_with_annotation_hg19_bed.lft.to.hg18.bed	SureSelect_All_Exon_50mb_with_annotation_hg19_bed
GENOME_SIZE	3,080,436,051	3,080,436,051	3,080,436,051	3,080,436,051	3,080,436,051
BAIT_TERRITORY	51,785,713	51,785,713	51,785,713	51,785,713	51,785,713
TARGET_TERRITORY	51,785,713	51,785,713	51,785,713	51,785,713	51,785,713
BAIT_DESIGN_EFFICIENCY	1	1	1	1	1
TOTAL_READS	86,737,493	118,031,729	73,829,463	74,374,761	243,388,612
PF_READS	86,737,493	118,031,729	73,829,463	74,374,761	243,388,612
PF_UNIQUE_READS	86,737,493	118,031,729	73,829,463	74,374,761	243,388,612
PCT_PF_READS	1	1	1	1	1
PCT_PF_UQ_READS	1	1	1	1	1
PF_UQ_READS_ALIGNED	78,714,486	107,269,121	67,063,728	67,517,987	217,982,059
PCT_PF_UQ_READS_ALIGNED	1	1	1	1	1
PF_UQ_BASES_ALIGNED	6,657,087,772	9,067,083,973	5,413,911,601	5,449,710,049	18,267,569,365
ON_BAIT_BASES	4,254,190,435	5,653,401,105	3,541,451,124	3,550,519,902	11,966,947,414
NEAR_BAIT_BASES	1,805,251,195	2,572,832,288	1,296,017,419	1,317,129,402	4,795,901,484
OFF_BAIT_BASES	597,646,142	840,850,580	576,443,058	582,060,745	1,504,720,467
ON_TARGET_BASES	4,254,190,435	5,653,401,105	3,541,451,124	3,550,519,902	11,966,947,414
PCT_SELECTED_BASES	0.910224	0.907263	0.893526	0.893194	0.917629
PCT_OFF_BAIT	0.089776	0.092737	0.106474	0.106806	0.082371
ON_BAIT_VS_SELECTED	0.702076	0.687241	0.732088	0.729412	0.713897
MEAN_BAIT_COVERAGE	82.149886	109.169127	68.386644	68.561765	231.085887
MEAN_TARGET_COVERAGE	86.286621	114.374236	71.781484	72.096659	236.214362
PCT_USABLE_BASES_ON_BAIT	0.57702	0.563498	0.564329	0.561626	0.578448
PCT_USABLE_BASES_ON_TARGET	0.57702	0.563498	0.564329	0.561626	0.578448
FOLD_ENRICHMENT	38.013239	37.088938	38.910994	38.75438	38.967707
ZERO_CVG_TARGETS_PCT	0.057893	0.054959	0.057756	0.06008	0.0233
FOLD_80_BASE_PENALTY	6.637432	7.14839	5.521653	5.545897	3.690849
PCT_TARGET_BASES_2X	0.915035	0.920331	0.916321	0.912459	0.973791
PCT_TARGET_BASES_10X	0.799733	0.81727	0.805502	0.797312	0.959696
PCT_TARGET_BASES_20X	0.711656	0.740977	0.703317	0.69474	0.941471
PCT_TARGET_BASES_30X	0.639944	0.681754	0.619312	0.612214	0.91584
